# Construction of Ketoenamine-Based Covalent Organic Frameworks with Electron-Rich Sites for Efficient and Rapid Removal of Iodine from Solution

**DOI:** 10.3390/molecules28248151

**Published:** 2023-12-18

**Authors:** Qi Tao, Xiao Zhang, Liping Jing, Lu Sun, Peipei Dang

**Affiliations:** 1 College of Energy and Electrical Engineering, Hohai University, Nanjing 211100, China; 2 Key Laboratory of Applied Surface and Colloid Chemistry Ministry of Education (MOE), School of Chemistry and Chemical Engineering, Shaanxi Normal University, Xi’an 710062, China; 3 College of Chemistry and Chemical Engineering, Qiqihar University, Qiqihar 161006, China; 4 Department of Chemistry, Northeast Normal University, Changchun 130024, China; 5 State Key Laboratory of Rare Earth Resource Utilization, Changchun Institute of Applied Chemistry, Chinese Academy of Sciences, Changchun 130022, China

**Keywords:** ketoenamine-based, covalent organic frameworks, adsorption, iodine

## Abstract

Porous covalent organic frameworks (COFs) have been widely used for the efficient removal of iodine from solution due to their abundance of electron-rich sites. In this study, two kinds of ketoenamine-based COFs, TpBD-(OMe)_2_ and TpBD-Me_2_, are successfully synthesized via Schiff base reaction under solvothermal conditions using 1, 3, 5-triformylphoroglucinol as aldehyde monomer, o-tolidine and o-dianisidine as amino monomers. The ability of TpBD-(OMe)_2_ and TpBD-Me_2_ to adsorb iodine in cyclohexane or aqueous solutions has been quantitatively analyzed and interpreted in terms of adsorption sites. TpBD-Me_2_ possesses two adsorption sites, -NH- and -C=O, and exhibits an adsorption capacity of 681.67 mg/g in cyclohexane, with an initial adsorption rate of 0.6 g/mol/min with respect to COF unit cell. The adsorption capacity of TpBD-(OMe)_2_ can be as high as 728.77 mg/g, and the initial adsorption rate of TpBD-(OMe)_2_ can reach 1.2 g/mol/min in the presence of oxygen atoms between the methyl group and the benzene ring. Compared with TpBD-Me_2_, the higher adsorption capacity and adsorption rate of TpBD-(OMe)_2_ towards iodine are not only reflected in organic solvents, but also in aqueous solutions. It is proven through X-ray photoelectron spectroscopy and Raman spectroscopy that iodine exists in the form of I_2_, I_3_^−^, and I_5_^−^ within TpBD-(OMe)_2_ and TpBD-Me_2_ after adsorption. This work not only expands the application of COFs in the field of iodine adsorption, but also provides research ideas and important an experimental basis for the optimization of iodine adsorption sites.

## 1. Introduction

The rapid development of the nuclear industry has gravely harmed the environment and posed a hazard to human health due to nuclear waste, making its removal an urgent issue that needs to be addressed [1,2,3]. The nuclear reaction process generates the radioactive isotopes ^129^I and ^131^I, ^129^I having a significantly longer half-life [2,4]. Iodine isotopes entering the human body without the right safeguards in place may lead to serious health conditions, such as acute radiation sickness and tumor formation [5,6]. In addition, iodine leakage into water bodies in the form of ions causes severe environmental pollution, while its solubility in organic solvents provides a considerable obstacle to industrial production and solvent recycling [7,8]. Therefore, the efficient removal of iodine from solution has emerged as a matter of widespread concern.

The commonly used iodine adsorbent consists of activated carbon impregnated with triethylenediamine (TEDA) or KI [9,10,11]. However, the accumulation of TEDA or KI within the adsorbents could lead to pore blockage in the activated carbon, thereby reducing their adsorption capacity over time [12]. Ag-based adsorbents include Ag-exchanged zeolites [13,14], titanosilicates [15], silver-doped porous oxide materials [16,17,18], silver-functionalized silica aerogels [19,20], and so on. The adsorption of iodine by silver-based adsorbents results in the formation of stable AgI [21,22], which exhibits low desorption efficiency when using suitable solvents for iodine, thereby limiting the recyclability of Ag-based adsorbents. The functional macroreticular resin exhibits a high specific surface area and exceptional stability [23,24]. Moreover, the amino, amide, and cationic components grafted onto the resin effectively enhance its adsorption capacity for iodine. Importantly, the absorbed iodine can be desorbed using an appropriate solvent, enabling the reuse of the resin material [24,25]. Newly developed metal organic frameworks (MOFs) [26,27,28,29] and porous organic polymers (POPs) [2,30,31,32,33] have gained significant attention due to their exceptional designability as monomers, high specific surface area, adjustable pore size, and pore volume, making them highly suitable for iodine adsorption in both vapor and solution systems. Covalent organic frameworks (COFs) connected by robust covalent bonds using multidentate organic building units emerge as significant adsorbents for iodine in solution owing to their excellent crystallinity, high specific surface area, one-dimensional open pores, low density, and exceptional thermal stability [34,35].

Considering the facile modifiability of COF monomers, electron-rich non-metallic heteroatoms, such as P, N, O, and S, were commonly incorporated into the skeleton of COFs. The incorporation of these elements effectively enhances the adsorption capacity of COFs towards iodine by leveraging the interaction between lone-pair electrons and iodine molecules [36,37,38,39,40]. The enol form of COFs, derived from the Schiff base reaction between 1, 3, 5-triformylphoroglucinol (Tp) and binary amino monomers, can undergo a conversion into a more thermodynamically stable ketone form, resulting in the formation of ketoenamine-based COFs [34]. The oxygen atom, as an electron-rich atom, is typically found in ketoenamine-based COFs in the form of a carbonyl or ether bond, which serve as important binding sites for iodine molecules. Since the oxygen in the ether bond possesses more lone electron pairs, its role in iodine adsorption cannot be overlooked. Current studies have primarily focused on investigating the influence of COFs’ pore size and specific surface area on their iodine adsorption performance [41], while there is a lack of clear experimental result to elucidate the impact of electron-rich sites on the adsorption performance. In this study, two types of COFs, namely TpBD-Me_2_ and TpBD-(OMe)_2_, were synthesized through the Schiff base reaction between Tp and o-tolidine (BD-Me_2_) and o-dianisidine (BD-(OMe)_2_), respectively. These COFs exhibit excellent crystallinity, high specific surface area, and remarkable thermal stability. We investigated their adsorption capacity and rate towards iodine in solution. It was observed that the presence of ether oxygen atoms in COFs led to enhanced adsorption capacity and faster adsorption kinetics for iodine uptake. This study provides new insights into understanding how TpBD-Me_2_ and TpBD-(OMe)_2_ interact with iodine at an atomic level while offering two novel materials for the efficient removal of iodine from solutions.

## 2. Results and Discussion

### 2.1. The Structure of TpBD-Me_2_ and TpBD-(OMe)_2_

**Figure 1:**
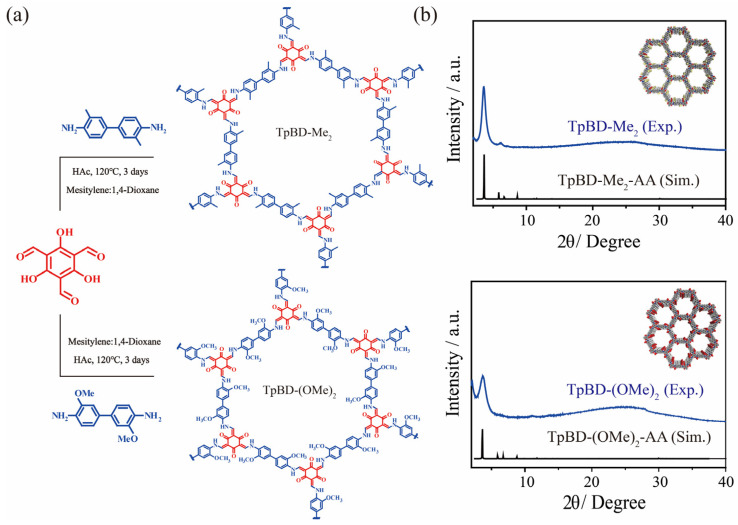
(**a**) Synthetic conditions and molecular structure of TpBD-Me_2_ and TpBD-(OMe)_2_; (**b**) PXRD results obtained from the experiments and simulations of TpBD-Me_2_ (**top**) and TpBD-(OMe)_2_ (**bottom**). Inset: AA stacking structural models of TpBD-Me_2_ and TpBD-(OMe)_2_.

**Scheme 1:**
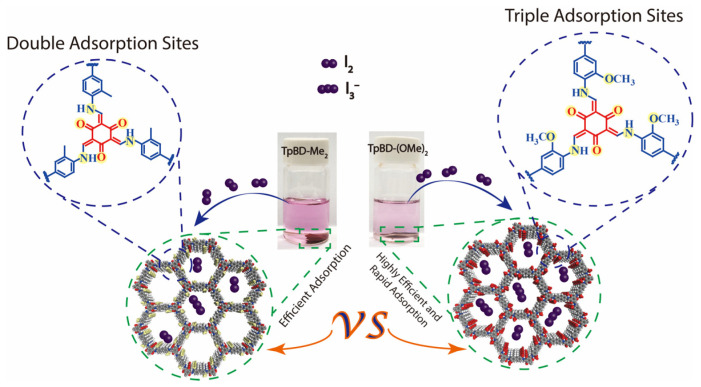
Two kinds of ketoenamine-based covalent organic frameworks (COFs) with electron-rich sites, namely TpBD-Me_2_ (double adsorption sites) and TpBD-(OMe)_2_ (triple adsorption sites) for iodine adsorption in solution.

To study the iodine adsorption performance of COFs from the perspective of adsorption sites, it is necessary to select suitable monomers to construct COFs in order to facilitate comparing the effects of differences in molecular structure on iodine adsorption. The structural differences between the already-reported TpBD-Me_2_ and TpBD-(OMe)_2_, which only exist in the oxygen atoms, allow us to simultaneously reveal the influence of both the ketoenamine-based skeletons and the ether oxygen atoms on iodine adsorption properties. The synthesis of TpBD-Me_2_ and TpBD-(OMe)_2_ was achieved by reacting Tp with BD-Me_2_ and BD-(OMe)_2_ (Figure 1a), respectively, under solvothermal conditions, as previously reported in the literature [34,38]. The molar ratio of Tp to BD-Me_2_ or BD-(OMe)_2_ in the reaction was 2:3, indicating that two molecules of Tp reacted with three molecules of BD-Me_2_ or BD-(OMe)_2_ to form a COF unit cell. TpBD-Me_2_ possesses two electron-rich functional groups consisting of secondary amine (-NH-) and carbyl oxygen (C=O), which serve as binding sites for iodine adsorption in solution. The incorporation of a methoxy component into the COF structure for TpBD-(OMe)_2_ additionally introduces ether oxygen sites (-O-), which could effectively enhance its adsorption capacity and rate of iodine (Scheme 1).

Before studying the iodine absorption property, we characterized the as-synthesized TpBD-Me_2_ and TpBD-(OMe)_2_ and compared the results with those in the literature to confirm their successful preparation. As shown in Figure 1b, the experimental powder X-ray diffraction (PXRD) results of TpBD-Me_2_ and TpBD-(OMe)_2_ are consistent with the theoretical simulations based on the AA stacking model using the Materials Studio 2020 software package, which can prove that they have good crystallinity. Fourier transform infrared spectroscopy (FT-IR) was employed for the characterization of the functional groups present in as-prepared TpBD-Me_2_ and TpBD-(OMe)_2_ (Figure S1). We carried out ^13^C solid-state nuclear magnetic resonance (NMR) spectroscopy to verify the atomic-level construction of as-prepared COFs (Figure S2). The thermal stability of the two types of COFs were assessed via thermogravimetric analysis in ambient air, revealing that TpBD-Me_2_ and TpBD-(OMe)_2_ exhibited notable thermal stability up to 300 °C without any discernible mass loss, which is comparable to that of other reported imine-based COFs [34,42] (Figure S3). The X-ray photoelectron spectroscopy (XPS) analysis of both types of COFs reveals the presence of C, N, and O elements (Figure S4, Table S1). The high-resolution XPS analysis of C element in TpBD-Me_2_ and TpBD-(OMe)_2_ reveals that the C1s signals can be categorized into three distinct carbon environments (Figure S5). The surface irregular flocculation morphology of TpBD-Me_2_ and TpBD-(OMe)_2_ was observed via scanning electron microscopy (SEM) and transmission electron microscopy (TEM) (Figures S6 and S7). In addition, it was demonstrated using PXRD that TpBD-Me_2_ and TpBD-(OMe)_2_ remained well stabilised in different solvents (Figure S8).

According to nitrogen adsorption-to-desorption isotherms (Figure S9a), both COFs exhibit a typical type I reversible isotherm, indicating significant nitrogen adsorption at low relative pressure and the existence of micropores in these materials. The N_2_ desorption curve of TpBD-(OMe)_2_ shows a slight hysteresis compared to TpBD-Me_2_, suggesting the presence of narrowly distributed mesopores. The Brunauer–Emmet–Teller (BET) plots to calculate surface area are shown in Figure S10. The surface area of TpBD-Me_2_ is 942.81 g/m^2^, surpassing that of TpBD-(OMe)_2_ (539.30 g/m^2^) (Table S2). The surface area of TpBD-Me_2_ is approximately twice as high as that of TpBD-(OMe)_2_, because integrating the ether oxygen atoms in the pore walls of the COFs further blocks the pores. The primary pore size of TpBD-Me_2_ is 1.83 nm, while that for TpBD-(OMe)_2_ is 1.88 nm, with a discernible presence of mesoporous regions (Figure S9b), which is consistent with the N_2_ desorption profile. Considering that the pore size of both COFs exceeds the molecular diameter of I_2_ (0.53 nm), the removal of I_2_ in solutions by TpBD-Me_2_ and TpBD-(OMe)_2_ could be attributed to chemisorption, while the influence of pore size on adsorption can be disregarded.

### 2.2. Adsorption of Iodine by TPBD-Me_2_ and TPBD-(OMe)_2_ in Cyclohexane

**Figure 2:**
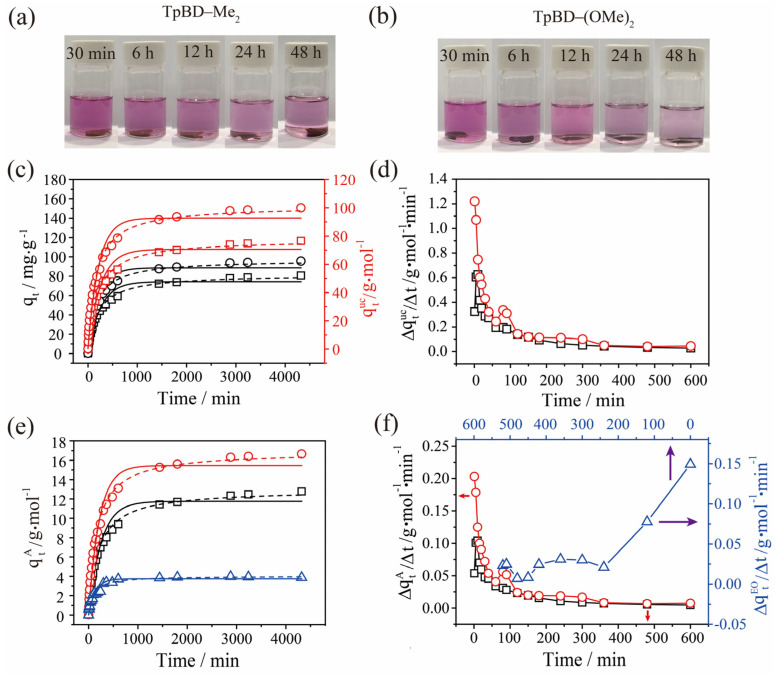
(**a**,**b**) The photographs of the process of I_2_ uptake after 10 mg TpBD-Me_2_ (**a**) and TpBD-(OMe)_2_ (**b**) were immersed in 10 mL of cyclohexane solution of I_2_ at the initial concentration of 100 mg·L^−1^; the incubation times are marked on the photos. (**c**) The values of $q_{t}$ (black) and $q_{t}^{uc}$ (red) were plotted against time for the adsorption kinetics of TpBD-Me_2_ (square) and TpBD-(OMe)_2_ (circle), which were fitted using pseudo-first-order (solid curves) and pseudo-second-order (dash curves) kinetic models. (**d**) Plots of the values of ${\Delta q}_{t}^{uc}/\Delta t$ for TpBD-Me_2_ (black square) and TpBD-(OMe)_2_ (red circle) in cyclohexane solution versus adsorption time. (**e**) Plots of the values of $q_{t}^{N+CO}$ (black square) for TpBD-Me_2_, $q_{t}^{N+O}$ (red circle) and $q_{t}^{EO}$ (blue triangle) for TpBD-(OMe)_2_ in cyclohexane solution versus adsorption time, which were fitted by pseudo-first-order (solid curves) and pseudo-second-order (dash curves) kinetic models. (**f**) Plots of the values of ${\Delta q}_{t}^{N+CO}/\Delta t$ (black square) for TpBD-Me_2_, ${\Delta q}_{t}^{N+O}/\Delta t$ (red circle), and ${\Delta q}_{t}^{EO}/\Delta t$ (blue triangle) for TpBD-(OMe)_2_ in cyclohexane solution versus adsorption time. The arrows are used to mark the horizontal and vertical coordinates of the corresponding curves.

To facilitate the separation and collection process, TpBD-Me_2_ or TpBD-(OMe)_2_ was compressed into a flaky shape for efficient iodine adsorption, and their morphologies are shown in Figure S11. The PXRD of the COFs in flake form is consistent with that of powders, indicating that they still maintain their original ordered structure (Figure S12). The thermogravimetric analysis of the flaky TpBD-Me and TpBD-(OMe)_2_ reveals that their decomposition temperatures remain above 300 °C, indicating that the applied pressure has not compromised their skeleton structure (Figure S13). The flecks of TpBD-Me_2_ and TpBD-(OMe)_2_ were immersed in a 100 mg/L iodine cyclohexane solution, respectively. Over time, the color intensity of the solutions gradually decreased. Notably, after 48 h of adsorption, it is evident that TpBD-(OMe)_2_ exhibits a significantly higher iodine absorption capacity compared to TpBD-Me_2_ (Figure 2a,b). UV-vis spectroscopy was used to monitor the concentration of iodine in cyclohexane to quantify the amount of iodine absorbed by COFs (Figure S14). A standard equation, based on UV-vis absorption values at various iodine concentrations, was employed to determine the concentration of iodine in cyclohexane solution during COF-mediated iodine uptake (Figure S15). The time-dependent change in adsorption capacity per unit mass ($q_{t}$) of 10 mg TpBD-Me_2_ and TpBD-(OMe)_2_ in a 100mg/L iodine cyclohexane solution is depicted in Figure 2c (Table S3). Adsorption equilibrium is achieved within approximately 30 h, with the adsorption capacity of TpBD-(OMe)_2_ for iodine being significantly higher compared to that of TpBD-Me_2_. The iodine absorption capacity of TpBD-(OMe)_2_ with respect to unit cell ($q_{t}^{uc}$) is significantly higher than that of TpBD-Me_2_, suggesting the crucial role played by the ether oxygen atoms in the process of iodine adsorption (Table S3). Based on the molar ratio of iodine molecule to COF unit cell ($q_{t}^{Mol}$), TpBD-(OMe)_2_ exhibits an additional adsorption capacity of 0.8 mol I_2_ per unit cell compared to TpBD-Me_2_ at equilibrium. The maximum adsorption capacities ($q_{e}$ and $q_{e}^{uc}$) at equilibrium were determined by simulating the values of $q_{t}$ and $q_{t}^{uc}$ using both pseudo-first-order and pseudo-second-order kinetic models (Tables S4 and S5). It is observed that the temporal changes in $q_{t}$ and $q_{t}^{uc}$ were better described by the pseudo-second-order kinetic model, suggesting a multi-site adsorption mechanism for iodine on the two COF materials, with charge transfer occurring between iodine molecules and adsorption sites. By calculating the adsorption rate of COFs of iodine (${\Delta q}_{t}^{uc}/\Delta t$), it is evident that the initial adsorption rate of TpBD-(OMe)_2_ (1.2 g/mol/min) exhibits a twofold increase compared to that of TpBD-Me_2_ (0.6 g/mol/min), while maintaining a consistent adsorption rate even after 100 min (Figure 2d).

By analyzing the iodine molecule binding sites within the crystal structure of both COFs, it is found that TpBD-Me_2_ possesses two distinct binding sites for iodine molecules (-NH-(N) and -C=O(CO)), while TpBD-(OMe)_2_ exhibits three binding sites (-NH-(N), -C=O(CO), and -O-Me (EO)). Each unit cell of the COFs contains six electron-rich sites of the same atoms for iodine adsorption. The value of $q_{t}^{uc}$ is divided by six to obtain the mass of adsorbed iodine per mole of adsorption sites ($q_{t}^{A}$) (Figure 2e). For TpBD-Me_2_, the adsorption capacity per mole of iodine binding site is represented by $q_{t}^{N+CO}$, whereas for TpBD-(OMe)_2_, it is denoted as $q_{t}^{N+O}$. By calculating the difference between $q_{t}^{N+O}$ and $q_{t}^{N+CO}$, the quantity of iodine adsorbed by each mole of oxygen atom in the ether bond ($q_{t}^{EO}$) could be determined (Table S6). According to experimental results and kinetic fitting, the maximum value of $q_{e}^{N+O}$ is greater than that of $q_{e}^{N+CO}$, indicating that the oxygen atoms in the methoxyl group play a crucial role in the iodine adsorption process (Table S7). The kinetic fitting results of $q_{t}^{N+O}$ and $q_{t}^{N+CO}$ are in better agreement with the pseudo-second-order kinetic model, providing further evidence for the multi-site adsorption nature of iodine molecules on COFs. Time-dependent calculation of the value of initial adsorption rate as for adsorption sites (${\Delta q}_{t}^{A}/\Delta t$) reveals that ${\Delta q}_{t}^{N+O}/\Delta t$ for TpBD-(OMe)_2_ at the three adsorption sites surpass that of TpBD-Me_2_ at the two sites, while the initial adsorption rate for oxygen atoms alone (${\Delta q}_{t}^{EO}/\Delta t$) exceeded that of TpBD-Me_2_ (Figure 2f). The above results demonstrate that the introduction of an oxygen atom not only enhances the iodine adsorption capacity, but also accelerates the adsorption rate. Although TpBD-Me_2_ has a higher surface area, its adsorption capacity and rate of iodine are lower than those of TpBD-(OMe)_2_, indicating that the adsorption site is the decisive factor in the process of the removal of iodine in solution.

**Figure 3:**
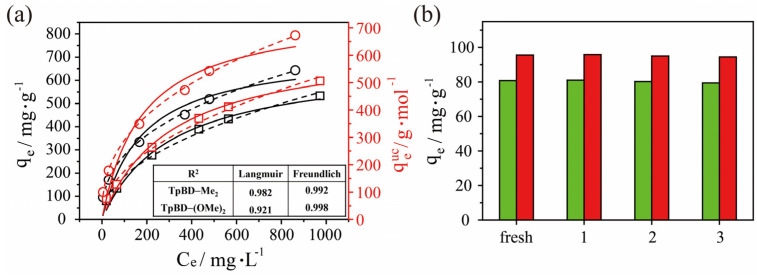
(**a**) Adsorption isotherms of TpBD-Me_2_ (square) and TpBD-(OMe)_2_ (circle) towards I_2_ in cyclohexane obtained by the values of $q_{e}\;$(black) and $q_{e}^{uc}$ (red) at different equilibrium I_2_ concentration ($C_{e}$), which were fitted by Langmuir (solid curves) and Freundlich models (dashed curves). Inset: correlation coefficient of adsorption isotherms fitted by Langmuir and Freundlich models for TpBD-Me_2_ and TpBD-(OMe)_2_. (**b**) Recyclability of TpBD-Me_2_ (green) and TpBD-(OMe)_2_ (red) for I_2_ adsorption in the cyclohexane solution at the initial concentration of 100 mg·L^−1^.

To further assess the iodine adsorption capacity of COFs, the iodine adsorption isotherm was determined by measuring the maximum amount of iodine adsorption at various equilibrium concentrations (Table S8, Figure 3a). Here, both the adsorption capacity with respect to the unit mass ($q_{e}$) and unit cell ($q_{e}^{uc}$) of these two COFs were compared, and Langmuir and Freundlich models were used to fit them (Tables S9 and S10). The maximum value of $q_{e}$ obtained by fitting the Langmuir model for TpBD-Me_2_ and TpBD-(OMe)_2_ are 681.67 mg/g and 728.77 mg/g, respectively. Additionally, the maximum values of $q_{e}^{uc}$ are determined to be 646.99 g·mol^−1^ and 761.68 g·mol^−1^, corresponding to 2.5 and 3.0 I_2_ per unit cell for TpBD-Me_2_ and TpBD-(OMe)_2_. The adsorption curve was fitted using the Langmuir equation with a correlation coefficient (R^2^) of 0.982 for TpBD-Me_2_ and 0.921 for TpBD-(OMe)_2_, while it was fitted using the Freundlich equation with a correlation coefficient (R^2^) of 0.992 for TpBD-Me_2_ and 0.998 for TpBD-(OMe)_2_. The adsorption data for the COF materials exhibit a higher degree of conformity with the Freundlich model rather than the Langmuir model, implying that iodine adsorption takes place in multiple layers within the pores of the COF materials. The value of 1/n fell within the range of 0.1 to 0.5 for Freundlich model, indicating a pronounced affinity between iodine molecules and the sites within the COF channels. The cyclability of COFs was evaluated by subjecting them to three cycles of elution using ethanol to achieve the complete desorption of the iodine molecule, followed by subsequent iodine adsorption in cyclohexane. The adsorption capacity of iodine in cyclohexane for TpBD-Me_2_ and TpBD-(OMe)_2_, as shown in Figure 3b, remains unchanged compared to the freshly prepared sample after three cycles, indicating their excellent cyclability and reusability.

**Figure 4:**
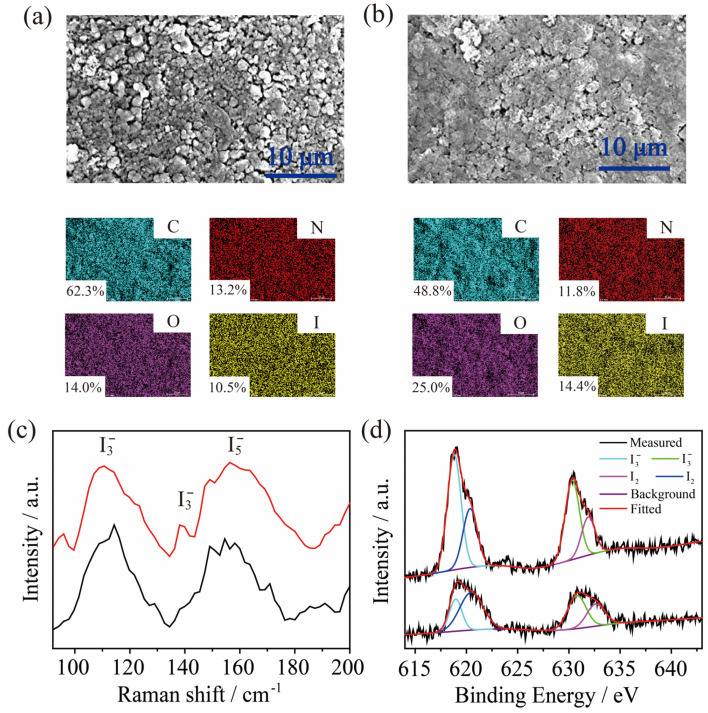
(**a**,**b**) SEM images and corresponding elemental mapping images of TpBD-Me_2_@I_2_ (**a**) and TpBD-(OMe)_2_@I_2_ (**b**) and atomic concentrations of different elements were marked on the photos. (**c**) Raman spectra of TpBD-Me_2_@I_2_ (black) and TpBD-(OMe)_2_@I_2_ (red) after adsorption equilibrium. (**d**) High-resolution XPS I3d spectrum of TpBD-Me_2_@I_2_ (bottom) andTpBD-(OMe)_2_@I_2_ (top). In (**a**–**d**), the I_2_ adsorption was carried out at the initial concentration of 500 mg·L^−1^ in cyclohexane solution.

The surface morphology of TpBD-Me_2_ and TpBD-(OMe)_2_ after iodine adsorption in cyclohexane (referred to as TpBD-Me_2_@I_2_ and TpBD-(OMe)_2_@I_2_) was observed using SEM, and their morphologies remain consistent with freshly prepared samples (Figure 4a,b). The PXRD patterns of TpBD-Me_2_@I_2_ and TpBD-(OMe)_2_@I_2_ show that they still have good crystallinity, indicating that the COF materials do not change their ordered structure after adsorption of iodine (Figure S16). The elemental distribution of TpBD-Me_2_@I_2_ and TpBD-(OMe)_2_@I_2_ was confirmed using energy dispersive spectroscopy (EDS). Notably, the variations in C, N, and O contents originate from the COFs themselves. Moreover, the higher iodine content observed in TpBD-(OMe)_2_@I_2_ compared to that in TpBD-Me_2_@I_2_ further substantiates the superior iodine absorption capacity of TpBD-(OMe)_2_ (Figure 4a,b). Raman spectroscopy was employed for the investigation of iodine molecule presence within COF channels (Figure 4c). The vibration peaks at 108 cm^−1^ and 140 cm^−1^ correspond to the symmetric and asymmetric stretching vibrations of I_3_^−^ ions, respectively. Additionally, a prominent peak at 165 cm^−1^ is observed, which corresponds to the stretching vibrations of I_5_^−^ (I_2_ + I_3_^−^) ions. The chemical state of iodine in COF material after iodine adsorption was further confirmed via X-ray photoelectron spectroscopy (XPS). The comparison of XPS spectra of TpBD-Me_2_ and TpBD-(OMe)_2_ before and after iodine uptake reveals that TpBD-Me_2_@I_2_ and TpBD-(OMe)_2_@I_2_ exhibit I 3d peaks at 631 eV and 620 eV, corresponding to I 3d_3/2_ and I 3d_5/2_, respectively, indicating the successful adsorption of iodine molecules (Figure S17). The atomic concentrations of various elements are presented in Table S11. Notably, the atomic concentration of iodine in TpBD-(OMe)_2_@I_2_ surpasses that of TpBD-Me_2_@I_2_, thereby reaffirming the superior adsorption capacity of TpBD-(OMe)_2_ towards iodine. The high-resolution XPS spectrum for I 3d reveals two splitting peaks in I 3d_3/2_ and I 3d_5/2_. The peaks with binding energies at 618.84 eV and 630.86 eV correspond to I_3_^−^, while the peaks at 620.39 eV and 632.40 eV represent I_2_ (Figure 4d). The XPS results for the analysis of chemical state of iodine in COF are consistent with those obtained from Raman spectroscopy.

**Figure 5:**
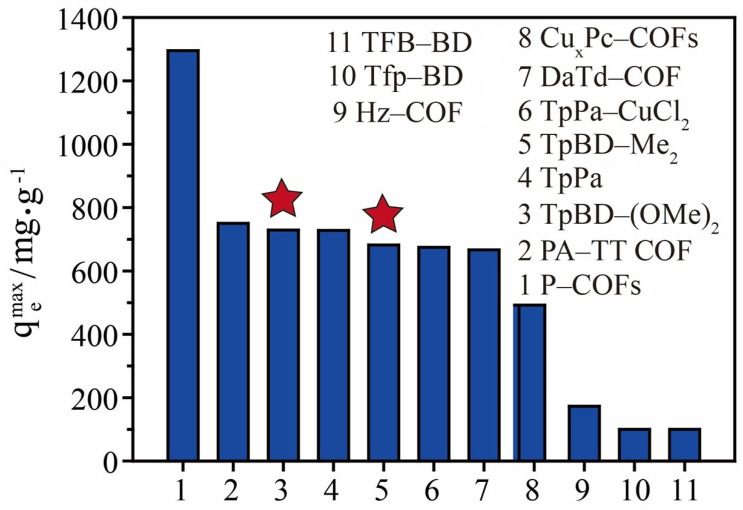
Comparison of maximum adsorption capacity ($q_{e}^{max}$) towards iodine for different COFs in organic solvent. Red stars are used to label TpBD-Me_2_ and TpBD-(OMe)_2_ prepared in this paper [36,39,40,43,44,45,46].

The iodine adsorption capacity of TpBD-Me_2_ and TpBD-(OMe)_2_ in cyclohexane is compared with the those reported in the literature for COFs [36,39,40,43,44,45,46,47], revealing that while their adsorption capacity does not reach the level of P-COF, they surpass most other COFs (Figure 5). The high adsorption capacity of TpBD-Me_2_ and TpBD-(OMe)_2_ could be attributed to their multiple binding sites for iodine molecules, as well as their large pore size and volume. Comparing TpBD-Me_2_ and TpBD-(OMe)_2_ with other porous materials, it is found that their iodine absorption capacity in organic solutions exceeds that of most MOFs and POPs (Table S12). Due to their inherent stability and recyclability as adsorption materials, COFs have been widely used in the field of iodine adsorption.

### 2.3. Adsorption of I_3_^−^ by TPBD-Me_2_ and TPBD-(OMe)_2_ in Aqueous Solution

**Figure 6:**
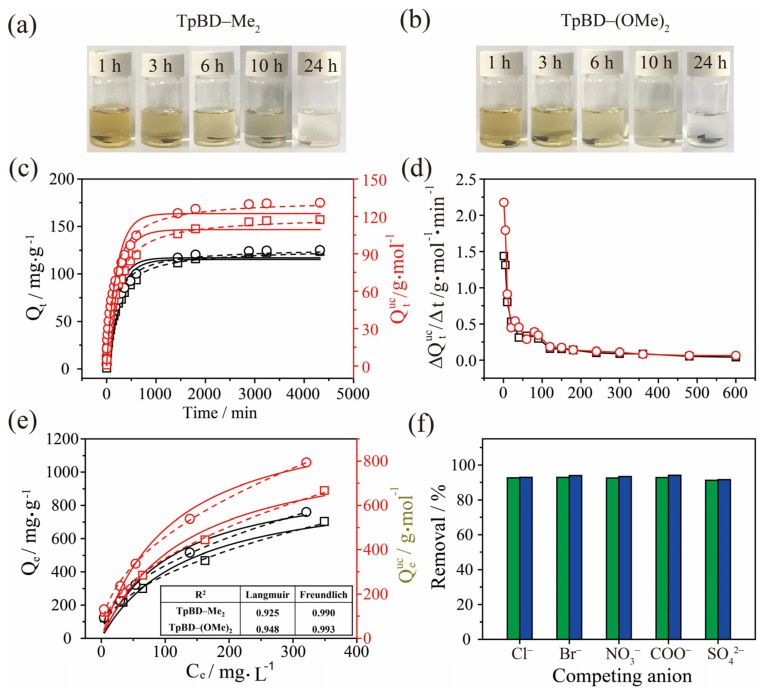
(**a**,**b**) The photographs of the process of I_3_^−^ uptake after 5 mg TpBD-Me_2_ (**a**) and TpBD-(OMe)_2_ (**b**) were immersed in 10 mL of aqueous solution of I_2_/KI at the initial concentration of 67 mg·L^−1^ of I_2_; the incubation times were marked on the photos. (**c**) The values of $Q_{t}\;$(black) and $Q_{t}^{uc}$ (red) were plotted against time for the adsorption kinetics of TpBD-Me_2_ (square) and TpBD-(OMe)_2_ (circle), which were fitted by pseudo-first-order (solid curves) and pseudo-second-order (dash curves) kinetics models. (**d**) Plots of the values of ${\Delta Q}_{t}^{uc}/\Delta t$ for TpBD-Me_2_ (black square) and TpBD-(OMe)_2_ (red circle) in aqueous solution versus adsorption time. (**e**) Adsorption isotherms of TpBD-Me_2_ (square) and TpBD-(OMe)_2_ (circle) towards I_2_ in KI/I_2_ aqueous solution obtained by the values of $Q_{e}$ (black) and $Q_{e}^{uc}$ (red) at different equilibrium I_2_ concentration ($C_{e}$), which were fitted by Langmuir (solid curves) and Freundlich models (dashed curves). Inset: correlation coefficient of adsorption isotherms fitted by Langmuir and Freundlich models for TpBD-Me_2_ and TpBD-(OMe)_2_. (**f**) Effect of typical competing anions on the removal percentage of iodine for TpBD-Me_2_ (green) and TpBD-(OMe)_2_ (blue) at an initial I_2_ concentration of 67 mg·L^−1^, in which the concentration of competing anions is 0.1 M.

Although I_2_ is poorly soluble in water, it can form I_3_^−^ with I^−^ ions, which in turn dissolves in water and becomes one of the major pollutants in water bodies. To expand the application scope of COF materials for solvent-based iodine adsorption, the iodine adsorption capacity of TpBD-Me_2_ and TpBD-(OMe)_2_ in aqueous solution was further investigated here. To enhance the solubility of iodine in water, I_2_ was dissolved in an aqueous solution containing KI (with a molar ratio of I_2_ to I^−^ as 1:5) to generate I_3_^−^ for subsequent adsorption. The flakes of TpBD-Me_2_ and TpBD-(OMe)_2_ were placed into the I_3_^−^ solution, and it was found that the solution became basically colorless after 24 h, indicating that both COFs possessed high removal efficiency for I_3_^−^ ions (Figure 6a,b). Depending on the concentration of iodine dissolved in the KI solution, the UV-vis spectrum of the I_3_^−^ solution was acquired and a standard curve was employed to quantify the iodine content in the solution (Figure S18). According to the standard curve, the quantity of iodine adsorbed by COF was determined based on the UV-vis absorbance of the solution (Figure S19). The temporal evolution of adsorption capacity per unit mass of COFs ($Q_{t}$) and per COF unit cells ($Q_{t}^{uc}$) towards I_2_ are depicted in Figure 6c (Table S13). The $Q_{t}$ and $Q_{t}^{uc}$ values of TpBD-Me_2_ and TpBD-(OMe)_2_ show a time-dependent increase, eventually reaching equilibrium. Notably, the $Q_{t}$ value of TpBD-(OMe)_2_ is slightly greater than that of TpBD-Me_2_, while the $Q_{t}^{uc}$ values display more pronounced differences between the two COFs. These findings suggest that TpBD-(OMe)_2_ offers enhanced advantages in adsorbing iodine from aqueous solution. The temporal evolutions of $Q_{t}$ and $Q_{t}^{uc}$ values exhibit greater conformity with the pseudo-second-order kinetic model, thereby further substantiating the existence of multiple iodine molecule binding sites within COFs and highlighting the occurrence of charge sharing and transfer between iodine molecules and these binding sites (Tables S14 and S15). The time-dependent iodine adsorption rate with respect to COF unit cell (${\Delta Q}_{t}^{uc}/\Delta t$) is presented in Figure 6d, where the initial adsorption rate of TpBD-(OMe)_2_ is twice as high as that of TpBD-Me_2_, and both rates reach equilibrium rapidly. It is noteworthy that both COFs exhibit a higher rate of iodine adsorption in water compared to cyclohexane, attributed to the poor solubility of iodine molecules in aqueous solutions.

The adsorption isotherms for iodine adsorption in aqueous solution by TpBD-Me_2_ and TpBD-(OMe)_2_ were determined based on the maximum values of $Q_{e}$ and $Q_{e}^{uc}$ at various equilibrium concentrations (Table S16, Figure 6e). The maximum iodine uptake of TpBD-Me_2_ and TpBD-(OMe)_2_, as determined by the Langmuir model, are 939.56 mg/g and 997.13 mg/g, respectively (Table S17). These values significantly exceed the iodine uptake in cyclohexane, indicating that water, despite being considered an undesirable solvent for iodine, exhibits a more favorable propensity for iodine adsorption. The maximum values of $Q_{e}^{uc}$ obtained from Langmuir model simulations for TpBD-Me_2_ and TpBD-(OMe)_2_ are 891.77 g/mol and 1042.15 g/mol, respectively, and TpBD-(OMe)_2_ exhibits a superior adsorption capability (Table S18). Based on the correlation coefficient (R^2^) obtained from the fitting analysis (Figure 6e), it is evident that the adsorption of iodine in water by COFs aligns more closely with the Freundlich model, implying a multilayered adsorption behavior of iodine molecules within the pores of COFs. Freundlich model simulations reveal that TpBD-Me_2_ and TpBD-(OMe)_2_ exhibit adsorption strengths of 1/n less than 0.5, thereby demonstrating the favorable affinity between the active adsorption sites in the COFs and iodine. Different types of ions tend to dissolve in water when COFs were employed for iodine uptake. However, the efficiency of iodine removal by the COFs in aqueous solutions is found to be unaffected by different anions, indicating that the presence of anionic species does not interfere with the iodine uptake ability of TpBD-Me_2_ and TpBD-(OMe)_2_ (Figure 6f and Figure S20).

**Figure 7:**
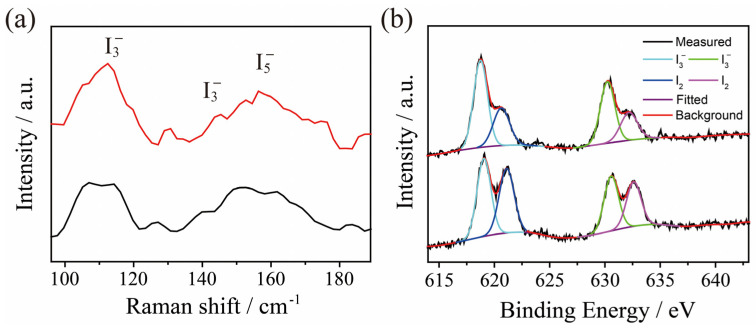
(**a**) Raman spectra of TpBD-Me_2_@I_3_^−^ (black) and TpBD-(OMe)_2_@I_3_^−^ (red) after adsorption equilibrium. (**b**) The high-resolution XPS spectrum of I 3d for TpBD-Me_2_@I_3_^−^ (bottom) and TpBD-(OMe)_2_@I_3_^−^ (top).

Raman spectroscopy and X-ray photoelectron spectroscopy were employed to confirm the presence of iodine species in TpBD-Me_2_ and TpBD-(OMe)_2_ after the adsorption of I_3_^−^ ions (designated as TpBD-Me_2_@I_3_^−^ and TpBD-(OMe)_2_@I_3_^−^) in an aqueous medium. The Raman spectrum reveals that the peaks observed at 108 cm^−1^ and 140 cm^−1^ correspond to the symmetric and asymmetric stretching vibrations of I_3_^−^, respectively, while the peaks detected at 160 cm^−1^ are attributed to the vibrations of I_5_^−^ (I_2_ + I_3_^−^) (Figure 7a). The two peaks observed at 632 eV and 620 eV in the XPS of TpBD-Me_2_@I_3_^−^ and TpBD-(OMe)_2_@I_3_^−^ correspond to I 3d_3/2_ and I 3d_5/2_, respectively (Figure S21). High-resolution XPS is able to separate I 3d_3/2_ and I 3d_5/2_ into two characteristic peaks, respectively, with the peaks at binding energies of 618.79 eV and 630.28 eV corresponding to I_3_^−^ and the peaks at binding energies of 620.67 eV and 632.28 eV corresponding to I_2_ (Figure 7b). The atomic concentrations of TpBD-Me_2_@I_3_^−^ and TpBD-(OMe)_2_@I_3_^−^ indicate that TpBD-(OMe)_2_ still exhibits a higher affinity for adsorbing I_3_^−^ ions (Table S19). The adsorption capacities of TpBD-Me_2_ and TpBD-(OMe)_2_ exhibit variations in different solvents, while the chemical state of iodine within the COF channels remains consistent.

## 3. Materials and Methods

### 3.1. Materials

2,4,6-Trihydroxylbenzene-1,3,5-tricarbaldehyde (Tp), o-Tolidine (BD-Me_2_), and o-Dianisidine (BD-(OMe)_2_) were purchased from Jilin Province Yanshen Technology Co., Ltd, Changchun, China. Iodine, Mesitylene, 1,4-dioxane, NaCl, NaBr, NaNO_3_, CH_3_COONa, and Na_2_SO_4_ were purchased from Sigma-Aldrich, Shanghai, China. EtOH, THF, DCM, Acetone, Cyclohexane, and CH_3_COOH were purchased from Beijing Chemical Industry Group Co. Ltd., Beijing, China. All the chemicals and materials were used without further purification. Water with resistivity of 18 MΩ·cm^−1^ was prepared using a Millipore Milli-Q system and used in all experiments.

### 3.2. Characterization

Powder X-ray diffraction (PXRD) was performed using D8 Advance X-ray Diffractometer (Bruker AXS, Karlsruhe, Germany) using Cu-Kα radiation (λ = 1.5418 Å), operating at 40 kV and 40 mA with a scanning rate of 1°·min^−1^ (2θ). FT-IR spectra was recorded on a Bruker IFS 66v/S Fourier transform infrared spectrometer. ^13^C NMR spectra were obtained on a Bruker Avance III model 400 MHz solid-state nuclear magnetic resonance (NMR) spectrometer at a magic angle spinning (MAS) rate of 5 kHz. X-ray photoelectron spectroscopy (XPS) was performed using a Thermo Scientific K-Alpha XPS (XPS, ESCALAB 250, Thermofisher, Waltham, U.S.) with a characteristic energy of 1486.7 eV. UV-vis absorption spectroscopy was performed by a UV-2450 spectrophotometer (HITACHI, Tokyo, Japan). Thermogravimetric analysis (TGA) was performed on a Netzch Sta 449c thermal analyzer system in the temperature range from 30 °C to 800 °C at a heating rate of 10 °C·min^−1^ in an air atmosphere. The N_2_ adsorption–desorption isotherms were measured on a Quantachrome Autosorb-iQ2 analyzer. For N_2_ adsorption measurements, the masses of TpBD-Me_2_ and TpBD-(OMe)_2_ used were 69.2 mg and 61.8 mg, respectively. Prior to measurements, the COFs were degassed under vacuum at 120 °C for 8 h in order to remove the gas adsorbed on the surface of the materials. Raman spectroscopy was performed on a Horiba LabRAM HR evolution instrument in the wavenumber range of 50–1000 cm^−1^ by using 633 nm laser (50 mW). The accumulation time of 600 s was applied to collect all the spectroscopic data. Scanning electron microscopy (SEM) imaging and energy dispersive spectroscopic (EDS) elementary mapping were performed on a Hitachi FE-SEM S-4800 scanning electron microscope with an acceleration voltage of 3 kV. The TEM images were observed on JEM-2100F, JEOL transmission electron microscopy.

### 3.3. Synthesis of TpBD-Me_2_ and TpBD-(OMe)_2_ Powders

In the typical synthesis, a Pyrex tube (o.d. × i.d. = 10 × 8 mm^2^ and length 18 cm) was charged with 0.2 mM 1,3,5-triformylphloroglucinol (Tp, 42.0 mg), 0.3 mM o-tolidine (BD-Me_2_, 63.7 mg), or o-dianisidine (BD-(OMe)_2_, 73.3 mg), 1.5 mL of mesitylene, 1.5 mL of 1,4-dioxane and 0.2 mL of 6 M aqueous acetic acid. This reaction mixture was sonicated for 10 min to obtain a homogeneous dispersion. After being frozen at 77 K (liquid N_2_ bath) and degassed by three freeze–pump–thaw cycles, the reaction tube was flame-sealed, reducing the total length by ca. 8.0 cm. Upon warming to room temperature, the reaction tube was then heated to 120 °C for 3 days. After cooling down to room temperature, the precipitates were isolated through filtration and washed with THF/DCM/acetone thrice. The products obtained were further purified through Soxhlet extraction using THF as the solvent for two days. After that, the products were dried in vacuo at 120 °C for 24 h to produce TpBD-Me_2_ in 90.4% yield and TpBD-(OMe)_2_ in 91.2% yield.

### 3.4. Study of Iodine Adsorption Property of TpBD-Me_2_ and TpBD-(OMe)_2_ in Cyclohexane

To avoid iodine sublimation, all iodine adsorption experiments in different solutions were carried out in dark brown bottles at room temperature. The absorption intensity of I_2_ cyclohexane solution with different concentrations of 5, 10, 25, 50, 100, 250, 500 mg/L was measured using UV-vis absorption spectroscopy, and the absorption intensity was plotted against the concentration to obtain the standard curve of I_2_ cyclohexane solution. After as-prepared TpBD-Me_2_ and TpBD-(OMe)_2_ were placed into the I_2_ cyclohexane solutions, the I_2_ concentrations in cyclohexane were monitored by means of UV-vis absorption spectroscopy. The I_2_ concentration in cyclohexane solutions at a given adsorption time (t, min) was calculated from the standard curve. The I_2_ adsorption capacity with respect to the mass of COFs ($q_{t}$, mg·g^−1^) and molar number of COF unit cells ($q_{t}^{uc}$, g·mol^−1^) and the ratio of the molar number of adsorbed I_2_ molecules to that of COF unit cells ($q_{t}^{mol}$) were calculated using Equations (1)–(3):

(1) $$q_{t}=\left(\left(C_{0}-C_{t}\right)V\right)/W_{COF}$$

(2) $$q_{t}^{uc}=\left(\left(C_{0}-C_{t}\right)V\right)/\left(W_{COF}/M_{COF}\right)$$

(3) $$q_{t}^{mol}=\left(\left(C_{0}-C_{t}\right)V/M_{I_{2}}\right)/\left(W_{COF}/M_{COF}\right)$$

where $C_{0}$ is the initial concentration of I_2_ cyclohexane solution, $C_{t}$ is the concentration of the solution at any time during the adsorption process, V is the volume of solution used for adsorption, $W_{COF}$ is the mass of the COFs and $M_{COF}$ is the molar mass of COF unit cell, which are 949.06 g/mol and 1045.06 g/mol, respectively, for TpBD-Me_2_ and TpBD-(OMe)_2_ according to the molar ratio of the reactive monomers. $M_{I_{2}}$ is the molar mass of the I_2_ molecule, the value of which is 253.8 g/mol.

The measured adsorption kinetics data of TpBD-Me_2_ and TpBD-(OMe)_2_ were fitted using pseudo-first-order and pseudo-second-order models, as shown in Equations (4) and (5), respectively.

(4) $$\ln\left(q_{e}-q_{t}\right)=lnq_{e}-k_{1}t$$

(5) $$t/q_{t}=1/k_{2}{\left(q_{e}\right)}^{2}+t/q_{e}$$

In these equations, $q_{t}$ and $q_{e}$ are the adsorption capacity at a given time (t, min) and at equilibrium state. When $q_{t}$ is the adsorption capacity with respect to COF unit cell ($q_{t}^{uc}$), $q_{e}$ is defined as adsorption capacity per molar number of COF unit cell ($q_{e}^{uc}$), and $k_{1}$ and $k_{2}$ are the pseudo-first-order rate constant (min^−1^) and the pseudo-second-order rate constant (g·mg^−1^·min^−1^), respectively.

When adsorption equilibrium is reached, adsorption capacity with respect to the mass of COFs ($q_{e}$) and the molar number of COF unit cell ($q_{e}^{uc}$), and the ratio of the molar number of adsorbed I_2_ molecules to that of COF unit cells ($q_{e}^{mol}$) at different initial concentrations ($C_{0}$) can be calculated using Equations (1)–(3) according to different equilibrium concentrations ($C_{e}$). The Langmuir isotherm is used to study the adsorption process occurring on the surface of the adsorbent (Equation (6)). In this equation, $K_{L}$ is the Langmuir constant, $q_{m}$ (mg·g^−1^, g·mol^−1^) is the maximum adsorption capacity, and $C_{e}$ (mg·L^−1^) is the iodine concentration at equilibrium state.

(6) $$q_{e}=q_{m}K_{L}C_{e}/(1+K_{L}C_{e})$$

The Freundlich isotherm is often used to study multi-layer adsorptions, as described in Equation (7),

(7) $$\ln q_{e}=1/n\ln C_{e}\;+\ln K_{F}$$

where 1/n represents the adsorption intensity (mg·L^−1^), $K_{F}$ is the Freundlich constant (mg·g^−1^, g·mol^−1^), and $C_{e}$ (mg·L^−1^) is the equilibrated concentration of iodine.

### 3.5. Study of Iodine Adsorption Property of TpBD-Me_2_ and TpBD-(OMe)_2_ in Aqueous Solution

The absorption intensity of KI/I_2_ aqueous solutions with different concentrations of I_2_ at 10, to 20, 30, 40, 50, 60, and 80 mg/L was measured using UV-vis absorption spectroscopy, and the absorption intensity was plotted against the concentration to obtain the standard curve. After as-prepared TpBD-Me_2_ and TpBD-(OMe)_2_ were placed into the KI/I_2_ aqueous solutions, the I_2_ concentration in the solution was monitored by means of UV-vis absorption spectroscopy. The I_2_ concentration in aqueous solutions at a given adsorption time (t, min) was calculated from the standard curve. The I_2_ adsorption capacity in KI/I_2_ aqueous solution with respect to the mass of COFs ($Q_{t}$, mg·g^−1^) and molar number of COF unit cells ($Q_{t}^{uc}$, g·mol^−1^) was calculated using Equations (1)–(3) using I_2_ concentration in solution at any time ($C_{t}$). The adsorption kinetics of I_2_ in aqueous solution can also be fitted through the pseudo-first-order and pseudo-second-order models (Equations (4) and (5)).

The maximum adsorption capacity towards I_2_ with respect to the mass of COFs ($Q_{e}$) and the molar number of COF unit cell ($Q_{e}^{uc}$) in KI/I_2_ aqueous solutions can also be calculated using Equations (1) and (2) according different I_2_ concentrations at equilibrium states ($C_{e}$). The adsorption isotherms could be simulated by Langmuir and Freundlich models to obtain the maximum adsorption capacity ($Q_{m}$) and the adsorption intensity (1/n) in aqueous solutions (Equations (6) and (7)).

### 3.6. Study of the Reusability of Iodine Uptake by TpBD-Me_2_ and TpBD-(OMe)_2_ in Cyclohexane

Freshly prepared TpBD-Me_2_ and TpBD-(OMe)_2_ were immersed in a cyclohexane solution containing 100 mg/L of I_2_ for three days to achieve adsorption equilibrium. Afterwards, TpBD-Me_2_ and TpBD-(OMe)_2_, which were saturated with adsorbed iodine, were immersed in ethanol for desorption. The ethanol was replaced every 12 h for a total of five cycles to ensure complete desorption of iodine molecules existed in the pores of COF materials. Next, TpBD-Me_2_ and TpBD-(OMe)_2_ were again immersed in 100 mg/L of I_2_ cyclohexane solution for I_2_ adsorption. This cycle was repeated 3 times, and the amount of iodine adsorbed by TpBD-Me_2_ and TpBD-(OMe)_2_ each time was compared with that of fresh preparation.

### 3.7. Evaluation of the Resistance to Anionic Interference on the Iodine Absorption Property in Aqueous Solutions

The KI/I_2_ solution, containing an I_2_ concentration of 67 mg/L, was supplemented with 0.1 M NaCl, NaBr, NaNO_3_, CH_3_COONa, and Na_2_SO_4_, respectively. Subsequently, 5 mg of TpBD-Me_2_ and TpBD-(OMe)_2_ were immersed in the aforementioned mixed solution. After a period of 3 days, the adsorption equilibrium was established, and the I_2_ concentration in the solution was determined using UV-vis spectroscopy. Subsequently, the adsorption capacity of iodine ($Q_{e}$) by COFs after mixing other anions in the KI/I_2_ solution was calculated based on Equation (1). The removal efficiency of iodine can be determined using Equation (8).

(8) $$Removal\;\%=Q_{e}/(C_{0}V)$$

## 4. Conclusions

The present study successfully synthesized two COFs, namely TpBD-Me_2_ and TpBD-(OMe)_2_, through the Schiff base reaction under solvothermal conditions. PXRD analysis, thermogravimetry, and N_2_ adsorption–desorption confirmed their high crystallinity, excellent thermal stability, large specific surface area, and presence of open pores. Considering the abundance of electron-rich N and O atoms in both COFs, their potential for efficient iodine absorption in cyclohexane and aqueous solution was explored. Both in water and in cyclohexane, TpBD-OMe_2_ exhibited higher iodine uptake and faster iodine uptake rates relative to TpBD-Me_2_, which was attributed to the existence of ether oxygen atoms increasing the number of its adsorption sites. The present study confirmed the crucial role of ether oxygen atoms in multiple COF monomers for iodine adsorption. Comparing the iodine adsorption of TpBD-(OMe)_2_ with other porous materials, it was found that its adsorption capacity was able to exceed that of most of the POPs (including COFs) and MOFs, making it a preferred material for iodine adsorption. Raman spectroscopy and XPS analysis revealed that iodine predominantly existed as monomeric species and their aggregates (I_2_, I_3_^−^, I_5_^−^) within the pores of the COFs. In this work, the adsorption capacities of TpBD-Me_2_ and TpBD-(OMe)_2_ in solutions were investigated for the first time from the perspective of adsorption sites, which improved the experimental basis and research ideas for the design of COF materials for iodine adsorption and provided new materials for the efficient removal of iodine from solutions.

## Data Availability

The data presented in this study are available on request from the corresponding author.

## Supplementary Materials

The following supporting information can be downloaded at: https://www.mdpi.com/article/10.3390/molecules28248151/s1, Figure S1: FT-IR spectra of COFs; Figure S2: Solid-state ^13^C CP-MAS NMR spectra of COFs; Figure S3: TGA profiles of COFs; Figure S4: XPS spectra of COFs; Figure S5: High-resolution XPS spectra of C1s; Figure S6: SEM images of COFs; Figure S7: TEM images of COFs; Figure S8: PXRD patterns of COFs in different solvents; Figure S9: Nitrogen adsorption-desorption isotherms curves and the pore size distribution of COFs; Figure S10: Plots of BET surface area of COFs; Figure S11: Photos of disk-shaped sheet of COFs; Figure S12: PXRD patterns of COF sheets; Figure S13: TGA profiles of COF sheets; Figure S14: Temporal absorption spectral evolution of cyclohexane solution of I_2_ during the adsorption process; Figure S15: UV-Vis spectra of cyclohexane solutions of I_2_ and plot of the absorption intensity versus the I_2_ concentration; Figure S16: PXRD patterns of COFs@I_2_; Figure S17: XPS spectra of COFs@I_2_; Figure S18: UV-vis spectra of I_3_^−^ aqueous solution and plot of the absorbance intensity of I_3_^−^ aqueous solution versus I_2_ concentration; Figure S19: Temporal absorption spectral evolution of aqueous solution of I_2_/KI during the adsorption process; Figure S20: Plots of the values of $Q_{e}$ in the presence of different kinds of competing anions; Figure S21: XPS spectra of COFs@I_3_^−^; Table S1: Summary of C, N, O atomic concentration of COFs; Table S2: Summary of the BET fitting results of nitrogen adsorption isotherms of COFs; Table S3: Summary of the values of $q_{t}$, $q_{t}^{uc}$ and $q_{t}^{Mol}$; Table S4: Summary of the experimental values of $q_{e}$ and calculated value of $q_{e}$ from fitting of their adsorption kinetics profiles; Table S5: Summary of the experimental values of $q_{e}^{uc}$ and the calculated value of $q_{e}^{uc}$ from fitting of their adsorption kinetics profiles; Table S6: Summary of the values of $q_{t}^{N+CO}$ of TpBD-Me_2_ and $q_{t}^{N+O}$, $q_{t}^{EO}$ of TpBD-(OMe)_2_; Table S7: Summary of the values of $q_{e}^{N+CO}$ of TpBD-Me_2_ and the values of $q_{e}^{N+O}$ and $q_{e}^{EO}$ of TpBD-(OMe)_2_ and the calculated value of $q_{e}^{N+CO}$, $q_{e}^{N+O}$ and $q_{e}^{EO}$ from fitting of their adsorption kinetics profiles; Table S8: Summary of the $q_{e}$, $q_{e}^{uc}$ and $q_{e}^{Mol}$ values at different initial I_2_ concentrations; Table S9: Summary of the fitting results of I_2_ adsorption isotherms in terms of $q_{e}$; Table S10: Summary of the fitting results of I_2_ adsorption isotherms in terms of $q_{e}^{uc}$; Table S11: Summary of the element atomic concentration of COFs@I_2_; Table S12: Summary of I_2_ adsorption capacity of reported porous materials in organic solvent; Table S13: Summary of the values of $Q_{t}$, $Q_{t}^{uc}$; Table S14: Summary of the experimental values of $Q_{e}$ and the calculated value of $Q_{e}$ from fitting of their adsorption kinetics profiles; Table S15: Summary of the experimental values of $Q_{e}^{uc}$ and the calculated value of $Q_{e}^{uc}$ from fitting of their adsorption kinetics profiles; Table S16: Summary of the $Q_{e}$ and $Q_{e}^{uc}$ values; Table S17: Summary of the fitting results of I_2_ adsorption isotherms in terms of $Q_{e}$; Table S18: Summary of the fitting results of I_2_ adsorption isotherms in terms of $Q_{e}^{uc}$; Table S19: Summary of the element atomic concentration of COFs@I_3_^−^. References [48,49,50,51,52,53,54,55,56,57,58,59,60,61,62,63,64,65,66,67,68,69,70,71,72,73,74,75,76] are cited in the supplementary materials.
