# Supplementary material for: Construction of Ketoenamine-Based Covalent Organic Frameworks with Electron-Rich Sites for Efficient and Rapid Removal of Iodine from Solution
Source: Molecules. 2023 Dec 18;28(24):8151. doi: 10.3390/molecules28248151 (PMC10745408; doi:10.3390/molecules28248151)
Supplement: Supplementary file 1 [file molecules-28-08151-s001.zip › molecules-2708405-supplementary.pdf]

# Supporting Information

## Contents


**1 Supporting Figures**..........2

**2 Supporting Tables**..........12

**References**..........21

# 1 Supporting Figures

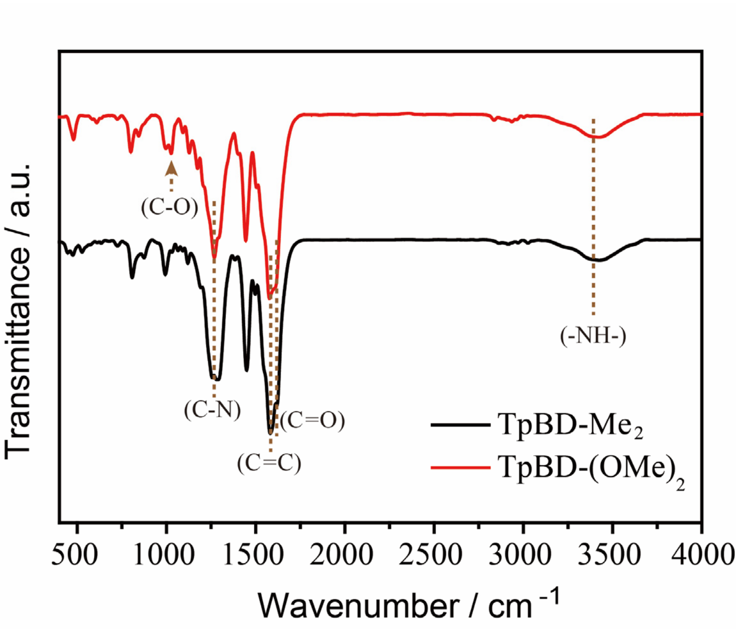


**Figure S1** The FT-IR spectra of TpBD-Me2 and TpBD-(OMe)2 and the characteristic peaks are marked on the photos.

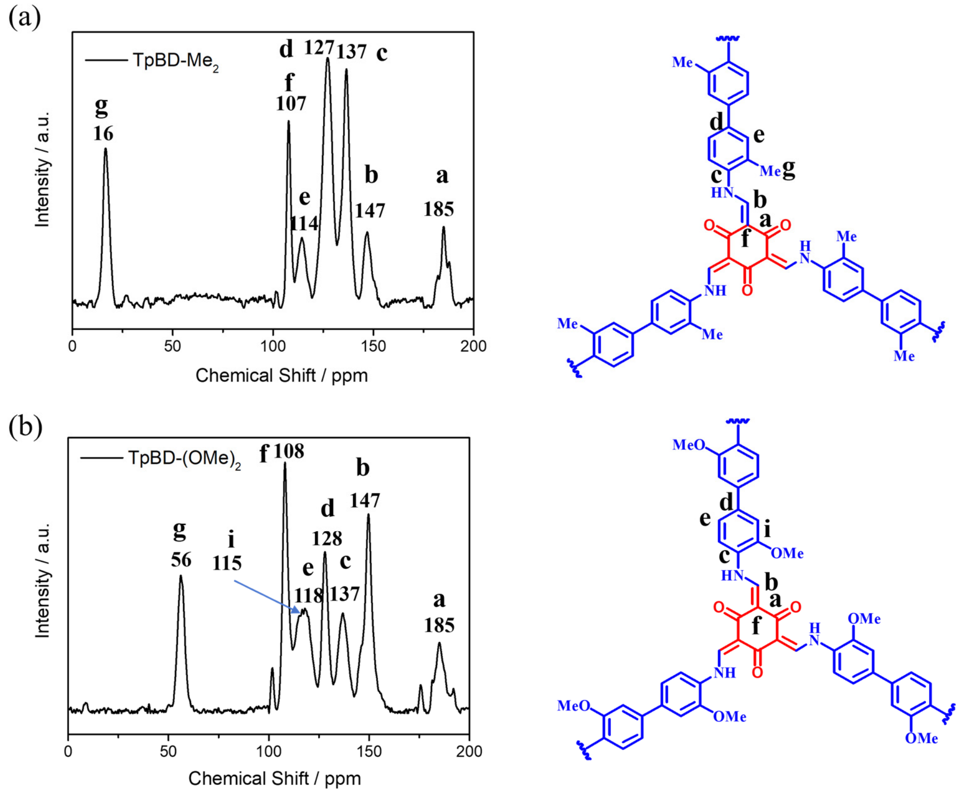


**Figure S2** Solid-state $^{13}C$ CP-MAS NMR spectra of TpBD-Me2 (a) and TpBD-(OMe)2 (b), in which the chemical shifts of carbon in different environments are marked in the photos.

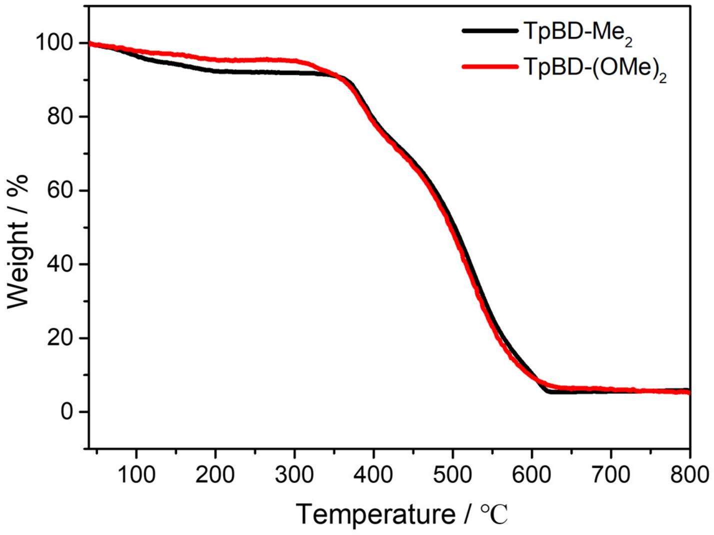


**Figure S3** Thermogravimetric analysis (TGA) profiles of TpBD-Me2 and TpBD-(OMe)2 obtained in the temperature range from 25°C to 800°C.

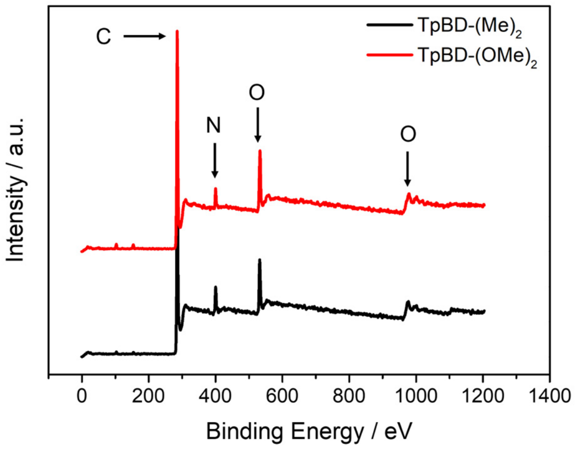


**Figure S4** XPS spectra of TpBD-Me2 (black), TpBD-(OMe)2 (red), in which the peaks of C, O and N are marked with arrows.

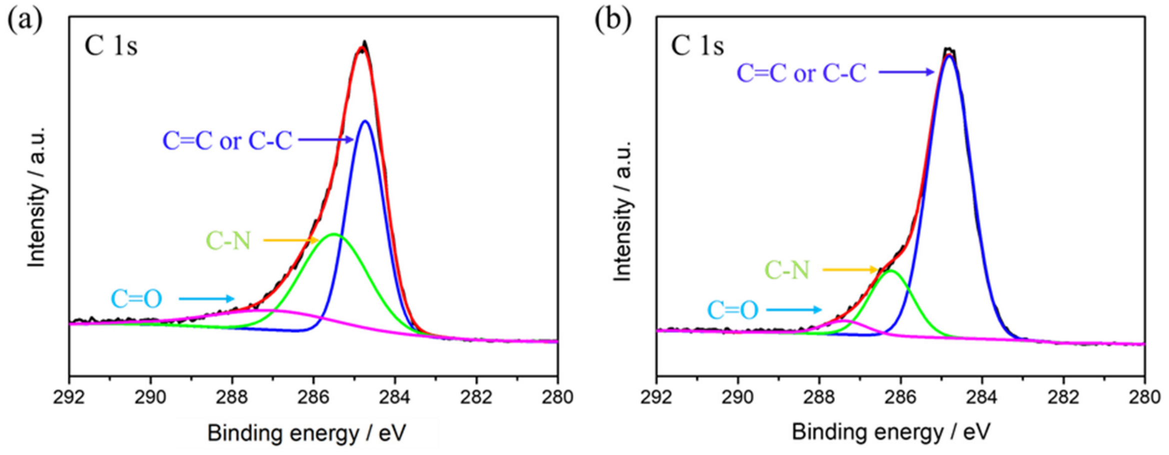


**Figure S5** High-resolution XPS spectra of C1s for TpBD-$Me_2$ (a) and TpBD-$(OMe)_2$ (b), which can be divided into three different carbon environments.

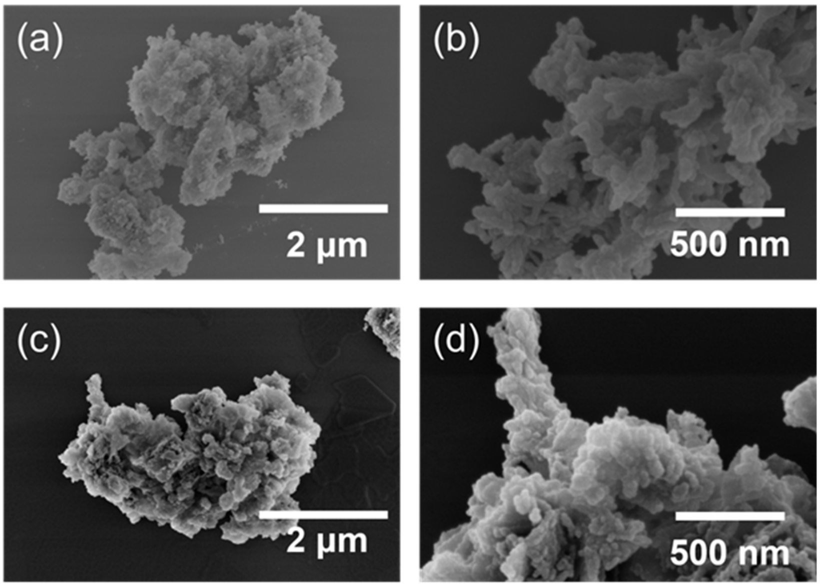


**Figure S6** Low (a,c) and high (b,d) magnification SEM images of TpBD-$Me_2$ (a,b) and TpBD-$(OMe)_2$ (c,d) powders.

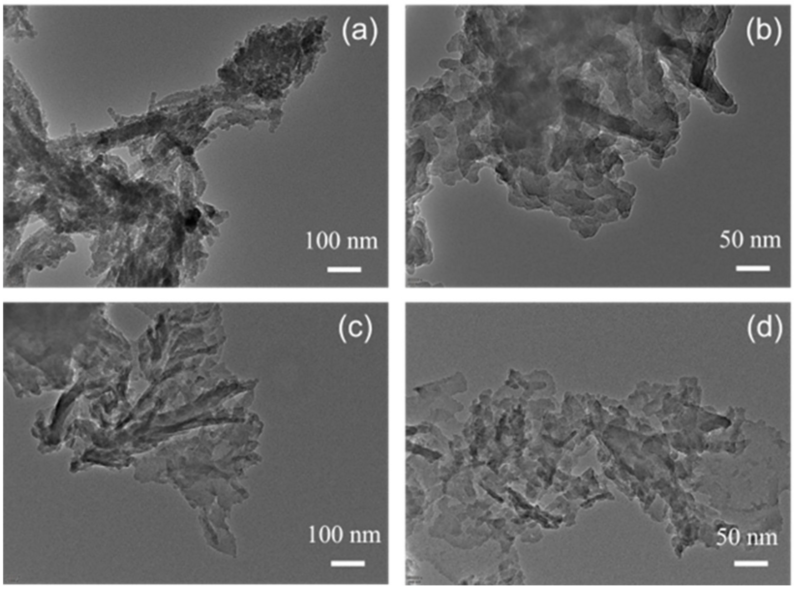


**Figure S7** The TEM images of TpBD-$Me_2$ (a,b) and TpBD-$(OMe)_2$ (c,d) powders with different magnifications.

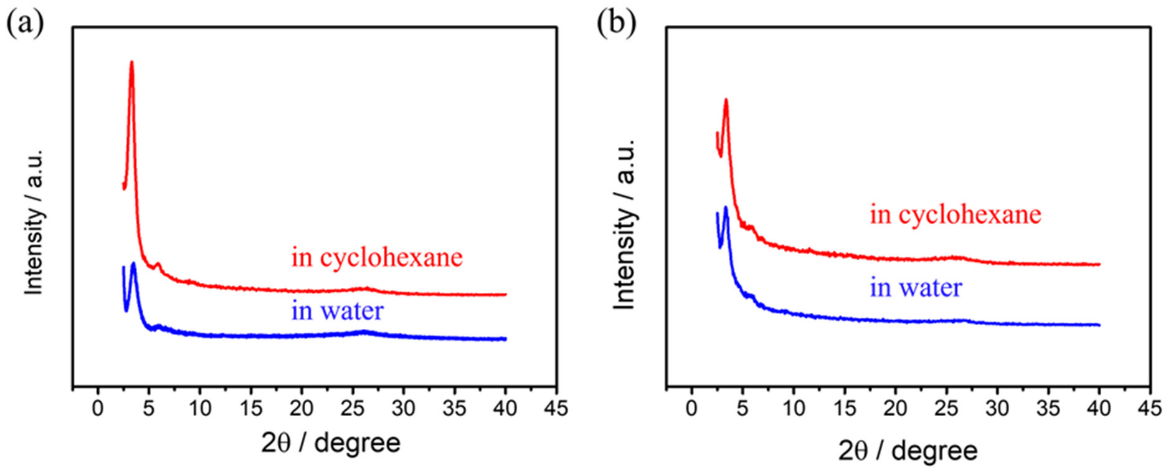


**Figure S8** PXRD patterns of TpBD-$Me_2$ (a) and TpBD-$(OMe)_2$ (b) powders immersing in water and cyclohexane for two weeks without refreshing solvent. Prior to PXRD measurements, the samples were separated via filtration and followed by drying under vacuum at 100°C for 48 hours.

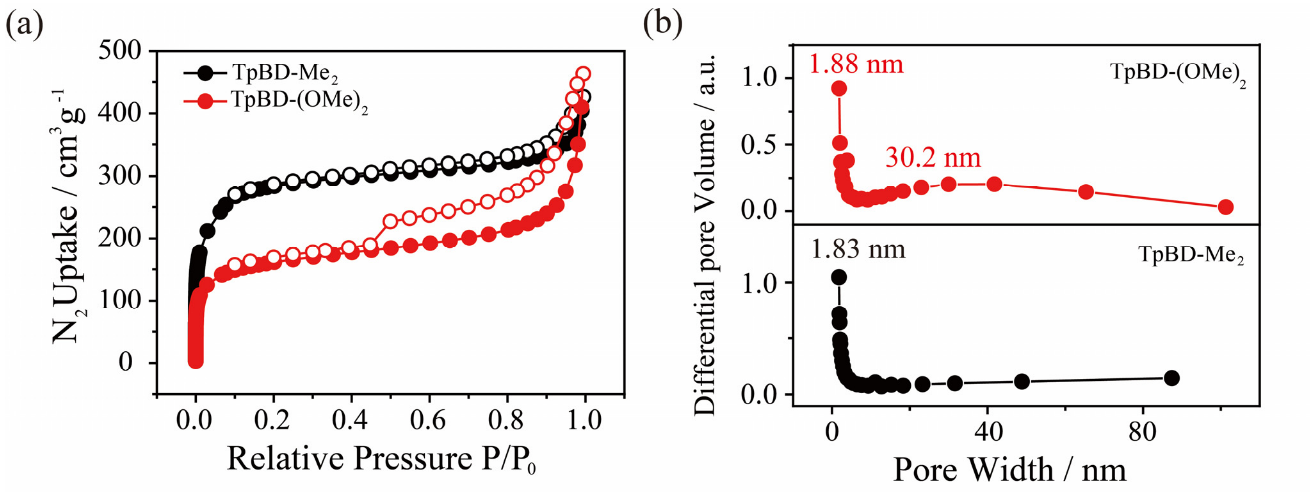


**Figure S9** (a) Nitrogen adsorption-desorption isotherms curves of TpBD-$Me_2$ and TpBD-$(OMe)_2$ (filled circles: adsorption, open circles: desorption) (b) The pore size distribution of TpBD-$Me_2$ and TpBD-$(OMe)_2$.

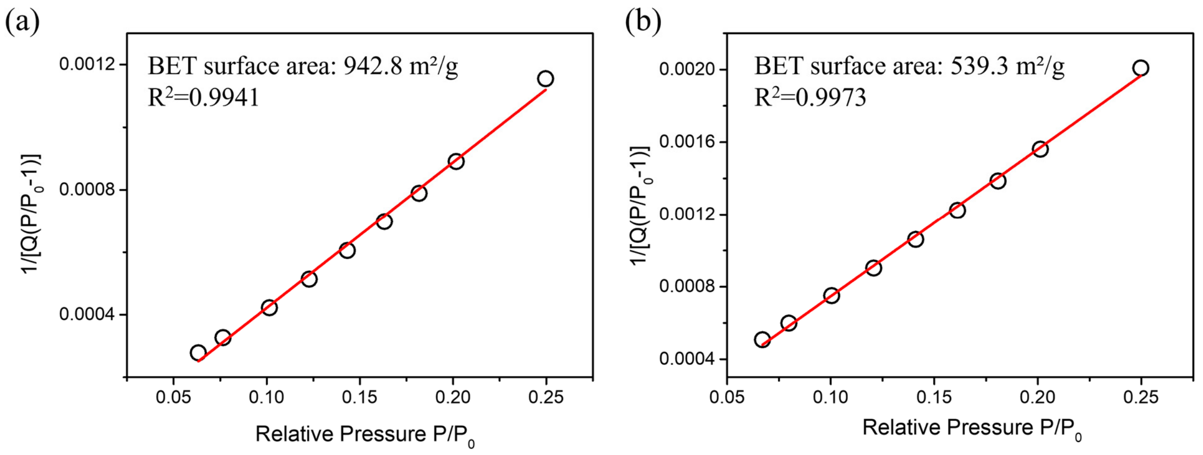


**Figure S10** Plots of BET surface area of TpBD-$Me_2$ (a) and TpBD-$(OMe)_2$ (b) obtained by in the range of relative pressure 0-0.25 and the calculated value of the specific surface area and the values of $R^2$ for linear fitting are marked on the photos.

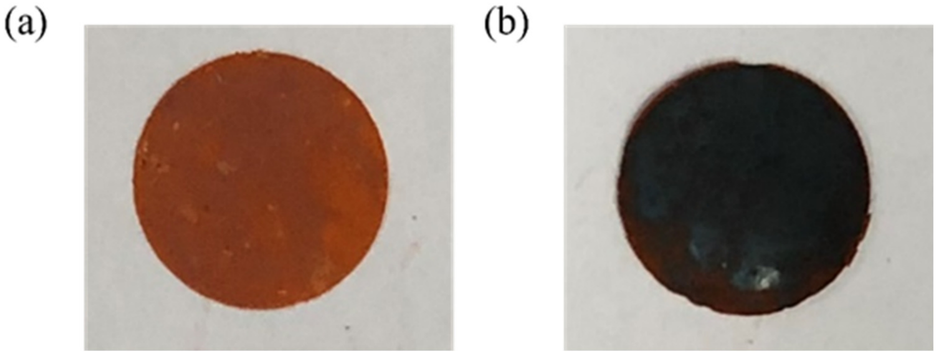


**Figure S11** Photos of as-prepared disk-shaped sheet of TpBD-$Me_2$ (a) and TpBD-$(OMe)_2$ (b) which were obtained by pressing the powders with a tablet press at a pressure of 10 MPa for 1 minute.

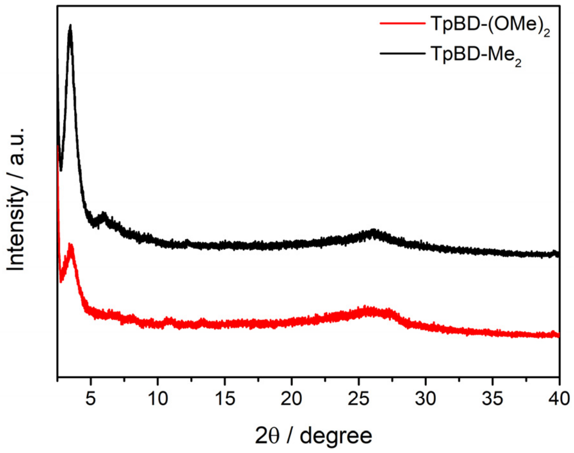


**Figure S12** PXRD patterns of as-prepared TpBD-$Me_2$ and TpBD-$(OMe)_2$ sheets after being pressed at a pressure of 10 MPa.

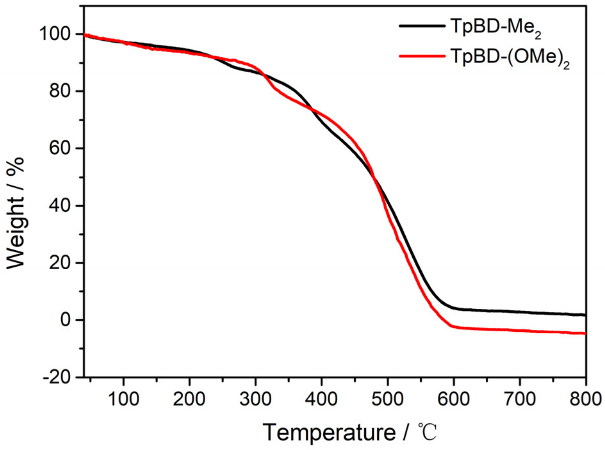

**Figure S13** Thermogravimetric analysis (TGA) profiles of TpBD-$Me_2$ and TpBD-$(OMe)_2$ sheets after being pressed at a pressure of 10 MPa.

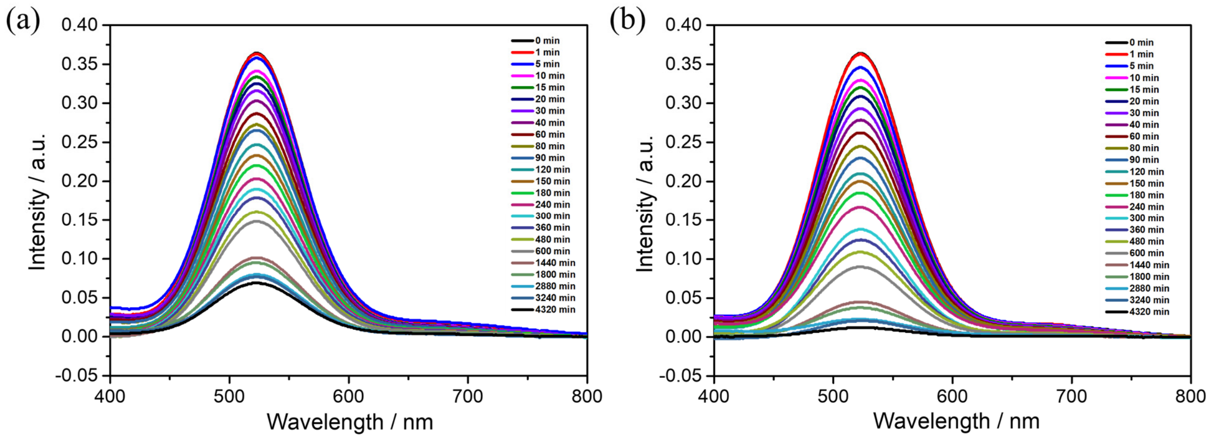


**Figure S14** Temporal absorption spectral evolution of 10 mL of cyclohexane solution of $I_2$ at the initial concentration of 100.25 mg·$L^{-1}$ recorded after 10 mg of TpBD-$Me_2$ (a) TpBD-$(OMe)_2$ (b) are placed inside.

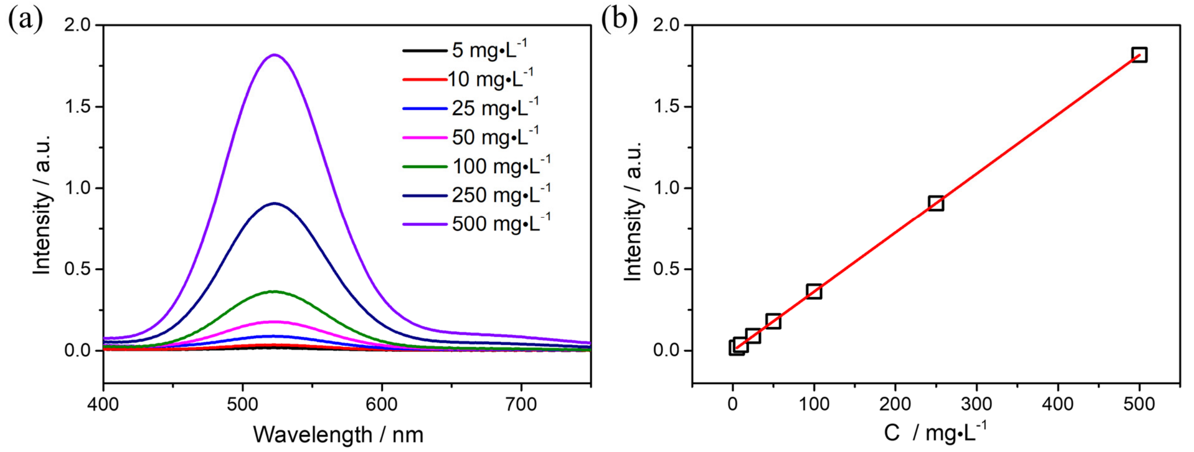


**Figure S15** (a) UV-Vis spectra of cyclohexane solutions of $I_2$ at different concentration ranging from 5, to 10, 25, 50, 100, 250 and 500 mg·$L^{-1}$.(b) Plot of the absorption intensity of $I_2$ at about 520 nm versus the $I_2$ concentration (C, mg·$L^{-1}$) in cyclohexane, which can be fitted according to a linear function of Intensity = 0.00364 - 0.0009065C with $r^2$ of 0.9999.

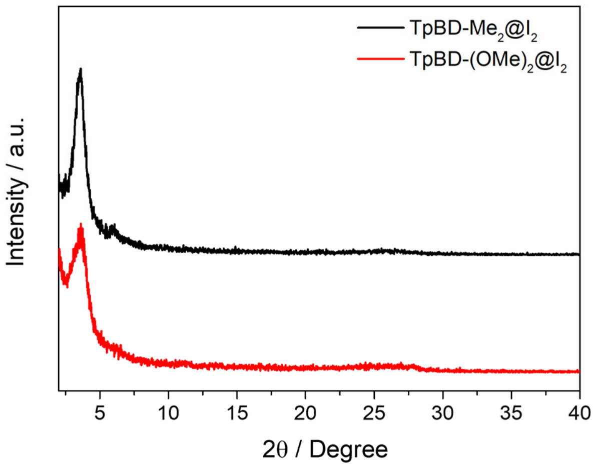


**Figure S16** PXRD patterns of TpBD-$Me_2$@$I_2$ (black) and TpBD-$(OMe)_2$@$I_2$ (red) after $I_2$ adsorption in cyclohexane at initial concentration of 100 mg·$L^{-1}$.

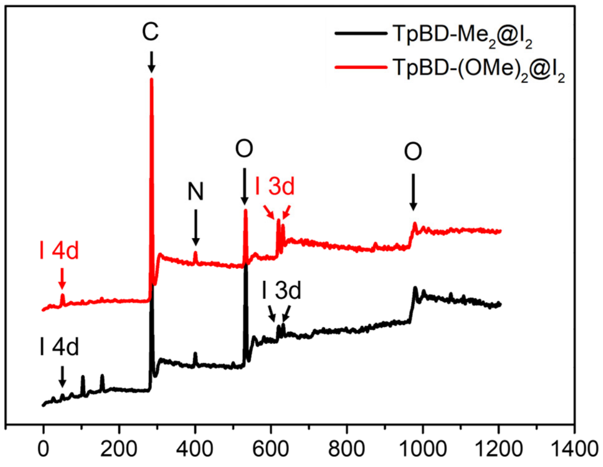


**Figure S17** XPS spectra of TpBD-$Me_2$@$I_2$ and TpBD-$(OMe)_2$@$I_2$ obtained by 72 h adsorption of TpBD-$Me_2$ and TpBD-$(OMe)_2$ in the cyclohexane solutions of $I_2$ at 500 mg·$L^{-1}$.

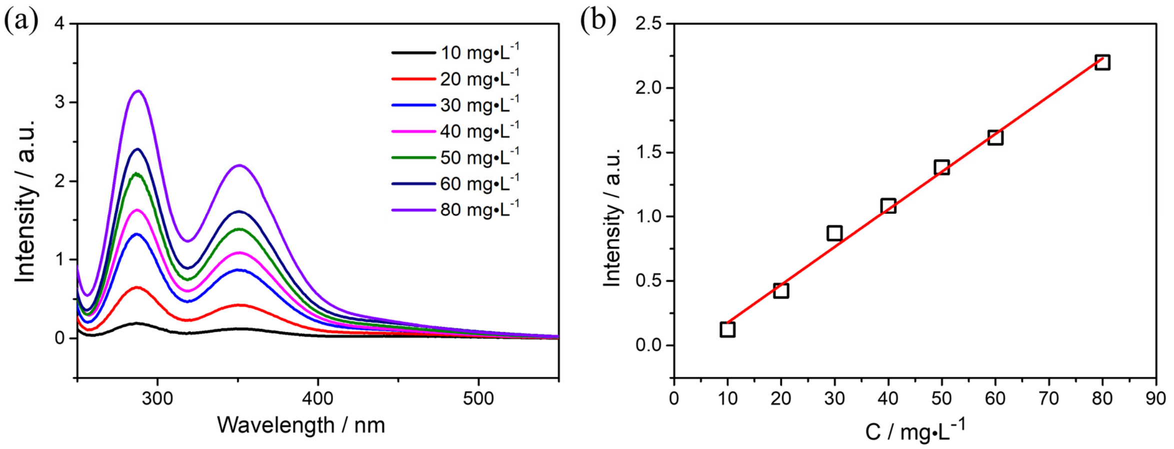


**Figure S18** (a) UV-vis spectra of $I_3^-$ aqueous solution (a mixture of KI and $I_2$ in the mole ration of 5:1) at concentration ranging from 10, to 20, 30, 40, 50, 60 and 80 mg·L$^{-1}$ of $I_2$. (b) Plot of the absorbance intensity of $I_3^-$ aqueous solution at 350 nm ($I_{350}$) versus $I_2$ concentration, which can be well fitted by a linear function, $I_{350} = 0.02935C_{I2}$ -0.1156 with $r^2$=0.992.

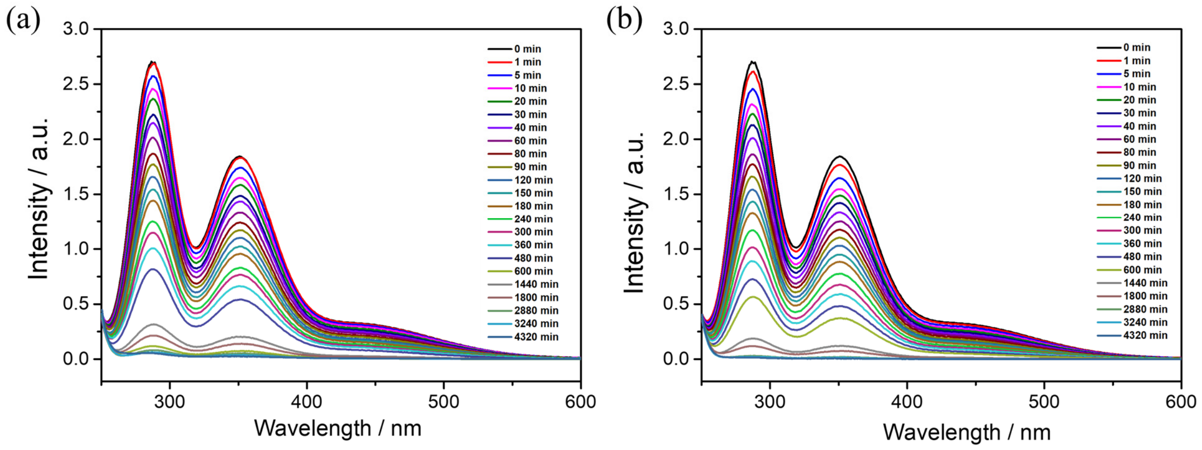


**Figure S19** Temporal absorption spectral evolution of 10 mL aqueous solution of $I_2$/KI at the initial concentration of 66.67 mg·L$^{-1}$ of $I_2$. recorded after 5 mg of TpBD-$Me_2$(a) TpBD-$(OMe)_2$(b) are placed inside.

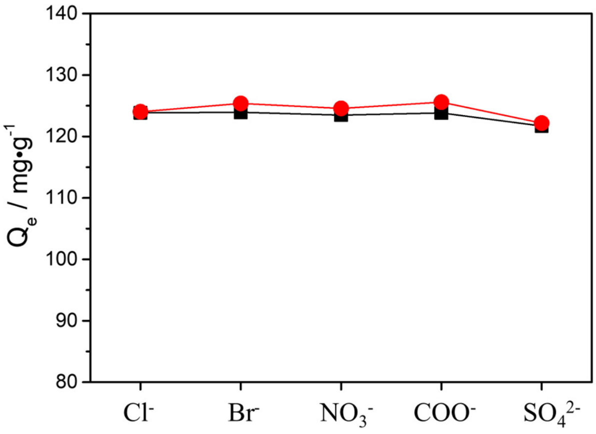


**Figure S20** Plot of the values of $Q_e$ of TpBD-Me2 (black square) and TpBD-(OMe)2 (red circle) in the presence of different kinds of competing anions, in which $I_2$ adsorption is carried out at the initial concentration of 67 mg·L$^{-1}$ of $I_2$ in $KI/I_2$ aqueous solution.

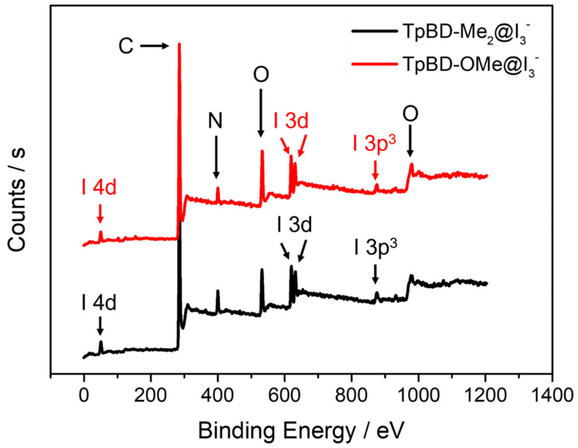


**Figure S21** XPS spectra of TpBD-Me2@$I_3^-$ (black) and TpBD-(OMe)2@ $I_3^-$ obtained by 72 h adsorption of TpBD-Me2 and TpBD-(OMe)2 in the $I_3^-$ aqueous solution (a mixture of KI and $I_2$ in the mole ration of 5:1) at the presence of 200 mg·L$^{-1}$ of $I_2$.

# 2 Supporting Tables

**Table S1** Summary of C, N, O atomic concentration of TpBD-$Me_2$ and TpBD-$(OMe)_2$ derived from the results of survey XPS spectra in Figure S4.

| COFs | C | O | N |
|---|---|---|---|
| TpBD-$Me_2$ | 81.11% | 10.49% | 8.40% |
| TpBD-$(OMe)_2$ | 82.69% | 11.87% | 5.44% |

**Table S2** Summary of the BET fitting results of nitrogen adsorption isotherms of TpBD-$Me_2$ and TpBD-$(OMe)_2$.

| COFs | Surface areas / $m^2·g^{-1}$ | Pore volume / $cm^3·g^{-1}$ | Pore diameter / nm |
|---|---|---|---|
| TpBD-$Me_2$ | 942.81 | 0.66 | 1.83 |
| TpBD-$(OMe)_2$ | 539.30 | 0.72 | 1.88 |

**Table S3** Summary of the values of $q_t$ , $q_t^{uc}$ and $q_t^{Mol}$ of TpBD-$Me_2$ and TpBD-$(OMe)_2$ obtained during 72 h (4320 min) adsorption of $I_2$ in cyclohexane.

| Time / min | TpBD-$Me_2$ | | | TpBD-$(OMe)_2$ | | |
|---|---|---|---|---|---|---|
| | $q_t$/mg·g$^{-1}$ | $q_t^{uc}$/g·moL$^{-1}$ | $q_t^{Mol}$ | $q_t$/mg·g$^{-1}$ | $q_t^{uc}$/g·moL$^{-1}$ | $q_t^{Mol}$ |
| 1 | 0.28 | 0.27 | 0.00105 | 0.28 | 0.29 | 0.00115 |
| 5 | 1.65 | 1.57 | 0.00617 | 4.95 | 5.17 | 0.0204 |
| 10 | 6.32 | 6.00 | 0.0236 | 9.34 | 9.76 | 0.0385 |
| 15 | 8.24 | 7.82 | 0.0308 | 12.09 | 12.64 | 0.0498 |
| 20 | 10.72 | 10.17 | 0.0401 | 15.11 | 15.79 | 0.0622 |
| 30 | 13.19 | 12.52 | 0.0493 | 19.51 | 20.39 | 0.0803 |
| 40 | 16.76 | 15.91 | 0.0627 | 23.35 | 24.40 | 0.0962 |
| 60 | 21.15 | 20.07 | 0.0791 | 28.02 | 29.29 | 0.115 |
| 80 | 25.00 | 23.73 | 0.0935 | 32.69 | 34.17 | 0.135 |
| 90 | 27.20 | 25.82 | 0.102 | 36.81 | 38.47 | 0.152 |
| 120 | 32.14 | 30.51 | 0.120 | 42.31 | 44.22 | 0.172 |
| 150 | 35.99 | 34.16 | 0.135 | 45.06 | 47.09 | 0.186 |
| 180 | 39.56 | 37.55 | 0.148 | 49.18 | 51.40 | 0.203 |
| 240 | 44.23 | 41.98 | 0.165 | 54.12 | 56.56 | 0.223 |
| 300 | 47.80 | 45.37 | 0.179 | 62.09 | 64.89 | 0.256 |
| 360 | 50.83 | 48.25 | 0.190 | 65.66 | 68.62 | 0.270 |

| | | | | | | |
|---|---|---|---|---|---|---|
| 480 | 55.77 | 52.93 | 0.209 | 70.06 | 73.22 | 0.289 |
| 600 | 59.34 | 56.32 | 0.222 | 75.28 | 78.68 | 0.310 |
| 1440 | 72.25 | 68.58 | 0.270 | 87.65 | 91.61 | 0.361 |
| 1800 | 73.90 | 70.14 | 0.276 | 89.56 | 93.60 | 0.369 |
| 2880 | 78.02 | 74.05 | 0.292 | 93.68 | 97.91 | 0.386 |
| 3240 | 78.85 | 74.84 | 0.295 | 94.23 | 98.48 | 0.388 |
| 4320 | 80.77 | 76.66 | 0.302 | 95.61 | 99.93 | 0.394 |

**Table S4** Summary of the experimental values of $q_e$ (exp. $q_e$) of TpBD-$Me_2$ and TpBD-$(OMe)_2$ obtained by 72 h adsorption of $I_2$ in cyclohexane at initial concentration of 100.25 mg·$L^{-1}$ and the calculated value of $q_e$ (cal. $q_e$) from fitting of their adsorption kinetics profiles according to pseudo-first-order or pseudo-second-order models. The kinetic parameters obtained by fitting according to pseudo-first-order or pseudo-second-order models are also listed in the Table S4.

| COFs | exp. $q_e$ /mg·$g^{-1}$ | Pseudo-first-order model | | | Pseudo-second-order model | | |
|---|---|---|---|---|---|---|---|
| | | cal. $q_e$ /mg·$g^{-1}$ | $k_1$/$min^{-1}$ | $R^2$ | cal. $q_e$ /mg·$g^{-1}$ | $k_2$/g·mg$^{-1}$·min$^{-1}$ | $R^2$ |
| TpBD-$Me_2$ | 80.77 | 74.34 | 0.004 | 0.969 | 81.95 | 6.316E-5 | 0.995 |
| TpBD-$(OMe)_2$ | 95.61 | 88.70 | 0.005 | 0.965 | 97.10 | 6.441E-5 | 0.993 |

**Table S5** Summary of the experimental values of $q_e^{uc}$ (exp. $q_e^{uc}$) of TpBD-$Me_2$ and TpBD-$(OMe)_2$ obtained by 72 h adsorption of $I_2$ in cyclohexane at initial concentration of 100.25 mg·$L^{-1}$ and the calculated value of $q_e^{uc}$ (cal. $q_e^{uc}$) from fitting of their adsorption kinetics profiles according to pseudo-first-order or pseudo-second-order models. The kinetic parameters obtained by fitting according to pseudo-first-order or pseudo-second-order models are also listed in the Table S5.

| COFs | exp. $q_e^{uc}$ /g·mol$^{-1}$ | Pseudo-first-order model | | | Pseudo-second-order model | | |
|---|---|---|---|---|---|---|---|
| | | cal. $q_e^{uc}$ /g·mol$^{-1}$ | $k_1$/$min^{-1}$ | $R^2$ | cal. $q_e^{uc}$ /g·mol$^{-1}$ | $k_2$/mol·g$^{-1}$·min$^{-1}$ | $R^2$ |
| TpBD-$Me_2$ | 76.66 | 70.56 | 0.004 | 0.969 | 77.78 | 6.655E-5 | 0.995 |
| TpBD-$(OMe)_2$ | 99.93 | 92.71 | 0.005 | 0.965 | 101.48 | 6.163 E-5 | 0.993 |

**Table S6** Summary of the values of $q_t^{N+CO}$ of TpBD-Me$_2$ and $q_t^{N+O}$, $q_t^{EO}$ of TpBD-(OMe)$_2$ obtained during 72 h (4320 min) adsorption of $I_2$ in cyclohexane.

| Time / min | TpBD-Me$_2$ $q_t^{N+CO}$/g·mol$^{-1}$ | TpBD-(OMe)$_2$ $q_t^{N+O}$/g·mol$^{-1}$ | $q_t^{EO}$/g·mol$^{-1}$ |
|---|---|---|---|
| 1 | 0.045 | 0.048 | 0.003 |
| 5 | 0.26 | 0.86 | 0.60 |
| 10 | 1.00 | 1.63 | 0.63 |
| 15 | 1.30 | 2.11 | 0.81 |
| 20 | 1.70 | 2.63 | 0.93 |
| 30 | 2.09 | 3.40 | 1.31 |
| 40 | 2.65 | 4.07 | 1.42 |
| 60 | 3.35 | 4.88 | 1.53 |
| 80 | 4.00 | 5.70 | 1.70 |
| 90 | 4.30 | 6.41 | 2.11 |
| 120 | 5.09 | 7.37 | 2.28 |
| 150 | 5.69 | 7.85 | 2.16 |
| 180 | 6.26 | 8.57 | 2.31 |
| 240 | 7.00 | 9.43 | 2.43 |
| 300 | 7.56 | 10.82 | 3.26 |
| 360 | 8.04 | 11.44 | 3.4 |
| 480 | 8.82 | 12.20 | 3.38 |
| 600 | 9.39 | 13.11 | 3.72 |
| 1440 | 11.43 | 15.27 | 3.84 |
| 1800 | 11.69 | 15.60 | 3.91 |
| 2880 | 12.34 | 16.32 | 3.98 |
| 3240 | 12.47 | 16.41 | 3.94 |
| 4320 | 12.78 | 16.66 | 3.88 |

**Table S7** Summary of the values of $q_e^{N+CO}$ (exp. $q_e^{N+CO}$) of TpBD-Me$_2$ and the values of $q_e^{N+O}$ and $q_e^{EO}$ (exp. $q_e^{N+O}$, $q_e^{EO}$) of TpBD-(OMe)$_2$ obtained by 72 h adsorption of $I_2$ in cyclohexane at initial concentration of 100.25 mg·L$^{-1}$ and the calculated value of $q_e^{N+CO}$, $q_e^{N+O}$ and $q_e^{EO}$ (cal. $q_e^{N+CO}$, $q_e^{N+O}$ and $q_e^{EO}$) from fitting of their adsorption kinetics profiles according to pseudo-first-order or pseudo-second-order models. The kinetic parameters obtained by fitting according to pseudo-first-order or pseudo-second-order models are also listed in the Table S7.

| COFs | exp. $q_e^{uc}$ /g·mol$^{-1}$ | Pseudo-first-order model | | | Pseudo-second-order model | | |
|---|---|---|---|---|---|---|---|
| | | cal. $q_e^{uc}$ /g·mol$^{-1}$ | $k_1$/min$^{-1}$ | $R^2$ | cal. $q_e^{uc}$ /g·mol$^{-1}$ | $k_2$/mol·g$^{-1}$·min$^{-1}$ | $R^2$ |
| $q_e^{N+CO}$ | 12.78 | 11.76 | 0.004 | 0.969 | 12.96 | 4.004E-4 | 0.995 |
| $q_e^{N+O}$ | 16.66 | 15.45 | 0.005 | 0.965 | 16.91 | 3.698 E-4 | 0.993 |
| $q_e^{EO}$ | 3.88 | 3.75 | 0.007 | 0.931 | 4.03 | 0.003 | 0.967 |

**Table S8** Summary of the $q_e$ (mg·g$^{-1}$), $q_e^{uc}$ (g·moL$^{-1}$) and $q_e^{Mol}$ values of TpBD-$Me_2$ and TpBD-$(OMe)_2$ COFs obtained by 72 h adsorption of $I_2$ in cyclohexane at different initial $I_2$ concentrations ($C_{I_2}^0$, mg·L$^{-1}$).

| $C_{I_2}^0$/ mg·L$^{-1}$ | TpBD-$Me_2$ | | | TpBD-$(OMe)_2$ | | |
|---|---|---|---|---|---|---|
| | $q_e$ /mg·g$^{-1}$ | $q_e^{uc}$ /g·mol$^{-1}$ | $q_e^{Mol}$ | $q_e$ /mg·g$^{-1}$ | $q_e^{uc}$ /g·mol$^{-1}$ | $q_e^{Mol}$ |
| 100.25 | 80.77 | 76.66 | 0.302 | 95.61 | 99.93 | 0.394 |
| 201.13 | 134.95 | 128.09 | 0.505 | 170.94 | 178.66 | 0.704 |
| 500.5 | 277.72 | 263.60 | 1.039 | 333.77 | 348.84 | 1.374 |
| 821.34 | 389.22 | 369.43 | 1.456 | 451.86 | 472.26 | 1.861 |
| 998.02 | 433.79 | 411.73 | 1.622 | 518.95 | 542.38 | 2.137 |
| 1505.58 | 533.10 | 505.99 | 1.994 | 643.54 | 672.60 | 2.650 |

**Table S9** Summary of the fitting results of $I_2$ adsorption isotherms in terms of $q_e$ (mg·g$^{-1}$) of TpBD-$Me_2$ and TpBD-$(OMe)_2$ in cyclohexane obtained by Table S8 according to Langmuir and Freundlich models.

| COFs | Langmuir model | | | Freundlich model | | |
|---|---|---|---|---|---|---|
| | $q_m$ /mg·g$^{-1}$ | $K_L$ /L·mg$^{-1}$ | $R^2$ | $K_F$ /mg$^{1-1/n}$g$^{-1}$L$^{1/n}$ | 1/n /mg·L$^{-1}$ | $R^2$ |
| TpBD-$Me_2$ | 681.67 | 0.003 | 0.982 | 20.97 | 0.474 | 0.992 |
| TpBD-$(OMe)_2$ | 728.77 | 0.006 | 0.921 | 45.98 | 0.390 | 0.998 |

**Table S10** Summary of the fitting results of $I_2$ adsorption isotherms in terms of $q_e^{uc}$ (g·mol$^{-1}$) of TpBD-$Me_2$ and TpBD-$(OMe)_2$ in cyclohexane obtained by Table S8 according to Langmuir and Freundlich models.

| COFs | Langmuir model | | | Freundlich model | | |
|---|---|---|---|---|---|---|
| | $q_m^{uc}$ /g·mol$^{-1}$ | $K_L$ /L·mg$^{-1}$ | $R^2$ | $K_F$ /gL$^{1/n}$ | 1/n /mg·L$^{-1}$ | $R^2$ |
| TpBD-$Me_2$ | 646.99 | 0.003 | 0.982 | 19.90 | 0.474 | 0.992 |
| TpBD-$(OMe)_2$ | 761.68 | 0.006 | 0.921 | 48.06 | 0.390 | 0.998 |

**Table S11** Summary of the element atomic concentration of TpBD-$Me_2$@$I_2$ and TpBD-$(OMe)_2$@$I_2$ after iodine adsorption derived from the results of survey XPS spectra in Figure S17.

| COFs | C | O | N | I |
|---|---|---|---|---|
| TpBD-$Me_2$@$I_2$ | 69.26% | 24.48% | 5.7% | 0.56% |
| TpBD-$(OMe)_2$ @$I_2$ | 65.36% | 30.17% | 3.65% | 0.82% |

**Table S12** Summary of $I_2$ adsorption capacity of reported porous materials in organic solvent.

| Types | Porous materials | adsorption capacity / mg·g$^{-1}$ | Reference |
|---|---|---|---|
| MOFs | UiO-66-PYDC | 1250 | S1 |
| | $[Cu_3(\mu_2\text{-}O)_2(p\text{-}tr_2Ph)_2$ $(HCOO)]$ $[NO_3]\cdot 3DMF\cdot 3H_2O$ | 1150 | S2 |
| | Th-BDAT | 1046 | S3 |
| | $[Eu_2(TATAB)_2]\cdot xDMF\cdot yH_2O$ | 758.72 | S4 |
| | Lac-Zn | 755 | S5 |
| | Th-TATAB | 750 | S6 |
| | $[Cu_2(TBDA\text{-}Cl)$ $(H_2O)\cdot 10DMF\cdot 30H_2O]_n$ | 548.2 | S7 |
| | Th-TTHA | 528 | S8 |
| | $[Co_2(H_2BATD)(DMF)_2]\cdot 2.5DMF\cdot 0.5H_2O$ | 480.99 | S9 |
| | Th-SINAP-8 | 473 | S10 |
| | SCNU-Z5 | 442 | S11 |
| | UiO-66 | 401 | S1 |
| | NS-1 | 320.5 | S12 |
| | MIL-101-$NH_2$ | 311 | S13 |
| | Th-UiO-66-$(NH_2)_2$ | 300 | S14 |
| | SCNU-Z4 | 237 | S15 |
| | NUC-3 | 212.04 | S16 |
| | MOF-5 | 155 | S17 |
| | [Cd (nds) $(bimh)_2$ · (bimh)]n | 148.5 | S18 |
| | U-TATAB | 146.72 | S19 |
| | CuAceAd crystals | 144 | S20 |
| | MIL-101(Cr) | 69.8 | S21 |
| POPs | Azo-Car-$CF_3$ | 1198.00 | S22 |
| | DTC-OP2 | 915.19 | S23 |

| | | | |
|---|---|---|---|
| | TTDP-3 | 784 | S24 |
| | TTDP-2 | 694.8 | S24 |
| | TTDP-1 | 682.6 | S24 |
| | P-TzTz | 568.28 | S25 |
| | DTC-OP3 | 528.45 | S23 |
| | TPAPyD-CMP | 333.8 | S26 |
| | NiP-CMP | 326 | S27 |
| | TPFM | 293.3 | S28 |
| | NRPOP-1 | 285.7 | S29 |
| | POBI | 239 | S30 |
| | NRPOP-2 | 212.8 | S29 |
| COFs | P-COFs | 1295.20 | S31 |
| | PA-TT COF | 750 | S32 |
| | **TpBD-(OMe)$_2$** | **728.77** | **This work** |
| | TpPa | 727.64 | S33 |
| | **TpBD-Me$_2$** | **681.67** | **This work** |
| | TpPa-CuCl$_2$ | 674.48 | S33 |
| | DaTd-COF | 666.79 | S34 |
| | Cu$_x$Pc-COFs | 492.27 | S35 |
| | Hz-COF | 173 | S36 |
| | Tfp-BD | 99.9 | S37 |
| | TFB-BD | 99.9 | S38 |

**Table S13** Summary of the values of $Q_t$ , $Q_t^{uc}$ of TpBD-Me$_2$ and TpBD-(OMe)$_2$ obtained during 72 h (4320 min) adsorption of $I_3^-$ in KI/$I_2$ aqueous solution.

| Time / min | TpBD-Me$_2$ | | TpBD-(OMe)$_2$ | |
|---|---|---|---|---|
| | $Q_t$/mg·g$^{-1}$ | $Q_t^{uc}$/mg·g$^{-1}$ | $Q_t$/mg·g$^{-1}$ | $Q_t^{uc}$/mg·g$^{-1}$ |
| 1 | 0.82 | 0.78 | 5.1 | 5.33 |
| 5 | 6.88 | 6.53 | 13.42 | 14.03 |
| 10 | 13.14 | 12.47 | 20.16 | 21.07 |
| 20 | 17.50 | 16.61 | 24.12 | 25.21 |
| 30 | 24.32 | 23.08 | 28.76 | 30.06 |
| 40 | 27.04 | 25.67 | 34.48 | 36.04 |
| 60 | 34.68 | 32.92 | 40.40 | 42.22 |
| 80 | 41.16 | 39.07 | 45.52 | 47.58 |

| | | | | |
|---|---|---|---|---|
| 90 | 45.86 | 43.53 | 50.42 | 52.70 |
| 120 | 50.62 | 48.05 | 55.46 | 57.96 |
| 150 | 55.94 | 53.10 | 60.98 | 63.73 |
| 180 | 60.50 | 57.42 | 65.42 | 68.37 |
| 240 | 69.10 | 65.59 | 72.70 | 75.98 |
| 300 | 73.38 | 69.65 | 79.52 | 83.11 |
| 360 | 80.40 | 76.31 | 85.32 | 89.17 |
| 480 | 88.58 | 84.08 | 92.66 | 96.84 |
| 600 | 93.82 | 89.05 | 100.10 | 104.62 |
| 1440 | 111.62 | 105.94 | 117.18 | 122.47 |
| 1800 | 115.9 | 110.01 | 120.48 | 125.92 |
| 2880 | 121.78 | 115.59 | 124.08 | 129.68 |
| 3240 | 122.78 | 116.54 | 124.7 | 130.33 |
| 4320 | 123.68 | 117.39 | 125.18 | 130.83 |

**Table S14** Summary of the experimental values of $Q_e$ (exp. $Q_e$) of TpBD-$Me_2$ and TpBD-$(OMe)_2$ obtained by 72 h adsorption of $I_3^-$ in KI/$I_2$ aqueous solution at initial concentration of 66.73 mg·$L^{-1}$ of $I_2$ and the calculated value of $Q_e$ (cal. $Q_e$) from fitting of their adsorption kinetics profiles according to pseudo-first-order or pseudo-second-order models. The kinetic parameters obtained by fitting according to pseudo-first-order or pseudo-second-order models are also listed in the Table S14.

| COFs | exp. $Q_e$ /mg·$g^{-1}$ | Pseudo-first-order model | | | Pseudo-second-order model | | |
|---|---|---|---|---|---|---|---|
| | | cal. $Q_e$ /mg·$g^{-1}$ | $k_1$/$min^{-1}$ | $R^2$ | cal. $Q_e$ /mg·$g^{-1}$ | $k_2$/$min^{-1}$ | $R^2$ |
| TpBD-$Me_2$ | 123.68 | 115.21 | 0.004 | 0.961 | 126.48 | 4.343E-5 | 0.992 |
| TpBD-$(OMe)_2$ | 125.18 | 117.01 | 0.005 | 0.938 | 127.36 | 5.249E-5 | 0.979 |

**Table S15** Summary of the experimental values of $Q_e^{uc}$ (exp. $Q_e^{uc}$) of TpBD-$Me_2$ and TpBD-$(OMe)_2$ obtained by 72 h adsorption of $I_3^-$ in KI/$I_2$ aqueous solution at initial concentration of 66.73 mg·$L^{-1}$ of $I_2$ and the calculated value of $Q_e^{uc}$ (cal. $Q_e^{uc}$) from fitting of their adsorption kinetics profiles according to pseudo-first-order or pseudo-second-order models. The kinetic parameters obtained by fitting according to pseudo-first-order or pseudo-second-order models are also listed in the Table S15.

| COFs | exp. $Q_e^{uc}$ /g·mol$^{-1}$ | Pseudo-first-order model cal. $Q_e$ /g·mol$^{-1}$ | $k_1$/min$^{-1}$ | $R^2$ | Pseudo-second-order model cal. $Q_e$ /mg·g$^{-1}$ | $k_2$/ mol·g$^{-1}$·min$^{-1}$ | $R^2$ |
|---|---|---|---|---|---|---|---|
| TpBD-$Me_2$ | 117.39 | 109.35 | 0.004 | 0.961 | 120.05 | 4.576E-5 | 0.991 |
| TpBD-$(OMe)_2$ | 130.83 | 122.30 | 0.005 | 0.938 | 133.10 | 5.023E-5 | 0.979 |

**Table S16** Summary of the $Q_e$ (mg·g$^{-1}$) and $Q_e^{uc}$ (g·moL$^{-1}$) values of TpBD-$Me_2$ and TpBD-$(OMe)_2$ obtained by 72 h adsorption of $I_3^-$ in in KI/$I_2$ aqueous solution at different initial $I_2$ concentrations ($C_{I_2}^0$ , mg·L$^{-1}$).

| $C_{I_2}^0$ | TpBD-$Me_2$ $Q_e$/mg·g$^{-1}$ | TpBD-$Me_2$ $Q_e^{uc}$/g·moL$^{-1}$ | TpBD-$(OMe)_2$ $Q_e$/mg·g$^{-1}$ | TpBD-$(OMe)_2$ $Q_e^{uc}$/g·moL$^{-1}$ |
|---|---|---|---|---|
| 66.73 | 123.68 | 117.39 | 125.18 | 130.83 |
| 142.52 | 216.94 | 205.91 | 225.80 | 235.99 |
| 214.82 | 300.18 | 284.92 | 322.48 | 337.04 |
| 396.51 | 468.24 | 444.43 | 515.96 | 539.26 |
| 700.72 | 703.24 | 667.48 | 759.80 | 794.10 |

**Table S17** Summary of the fitting results of $I_2$ adsorption isotherms in terms of $Q_e$ (mg·g$^{-1}$) of TpBD-$Me_2$ and TpBD-$(OMe)_2$ COFs in KI/$I_2$ aqueous solution obtained by Table S16 according to Langmuir and Freundlich models.

| COFs | Langmuir model $Q_m$ /mg·g$^{-1}$ | $K_L$ /L·mg$^{-1}$ | $R^2$ | Freundlich model $K_F$ /mg$^{1-1/n}$g$^{-1}$L$^{1/n}$ | 1/n /mg·L$^{-1}$ | $R^2$ |
|---|---|---|---|---|---|---|
| TpBD-$Me_2$ | 939.56 | 0.007 | 0.925 | 41.44 | 0.481 | 0.990 |
| TpBD-$(OMe)_2$ | 997.13 | 0.009 | 0.948 | 49.77 | 0.472 | 0.993 |

**Table S18** Summary of the fitting results of $I_2$ adsorption isotherms in terms of $Q_e^{uc}$ (g·mol$^{-1}$) of TpBD-$Me_2$ and TpBD-$(OMe)_2$ COFs in KI/$I_2$ aqueous solution obtained by Table S16 according to Langmuir and Freundlich models.

| COFs | Langmuir model | | | Freundlich model | | |
| --- | --- | --- | --- | --- | --- | --- |
| | $Q_m^{uc}$ /g·mol$^{-1}$ | $K_L$ /L·mg$^{-1}$ | $R^2$ | $K_F$ /gL$^{1/n}$ | 1/n /mg·L$^{-1}$ | $R^2$ |
| TpBD-$Me_2$ | 891.77 | 0.007 | 0.925 | 39.34 | 0.481 | 0.990 |
| TpBD-$(OMe)_2$ | 1042.15 | 0.009 | 0.948 | 52.02 | 0.472 | 0.993 |

**Table S19** Summary of the element atomic concentration of TpBD-$Me_2$@$I_3^-$ and TpBD-$(OMe)_2$ @$I_3^-$ after $I_3^-$ adsorption derived from the results of survey XPS spectra in Figure S21.

| COFs | C | O | N | I |
| --- | --- | --- | --- | --- |
| TpBD-$Me_2$@$I_3^-$ | 79.8% | 11.02% | 8.13% | 1.05% |
| TpBD-$(OMe)_2$ @$I_3^-$ | 80.79% | 11.93% | 5.76% | 1.52% |

# References

[S1] Wang, Z.; Huang, Y.; Yang, J.; Li, Y.; Zhuang, Q.; Gu, J. The Water-Based Synthesis of Chemically Stable Zr-Based MOFsUsing Pyridine-Containing Ligands and Their Exceptionally High Adsorption Capacity for Iodine. *Dalton Trans.* **2017**, 46, 7412-7420.

[S2] Xu, T.; Li, J.; Jia, M.; Li, G.; Liu, Y. Contiguous Layer Based Metal–Organic Framework with Conjugated π-Electron Ligand for High Iodine Capture. *Dalton Trans.* **2021**, 50, 13096-13102.

[S3] Ju, Y.; Li, Z.; Qiu, J.; Li, X.; Yang, J.; Zhang, Z.; He, M.; Wang, J.; Lin, J. Adsorption and Detection of Iodine Species by a Thorium-Based Metal−Organic Framework. *Inorg. Chem.* **2023**, 62, 8158-8165.

[S4] Zhao, Y.; Zhang, N.; Wang, Y.; Bai, F.; Xing, Y.; Sun, L. Ln-MOFs with Window-Shaped Channels Based on Triazine Tricarboxylic Acid as a Linker for the Highly Efficient Capture of Cationic Dyes and Iodine. *Inorg. Chem. Front.* **2021**, 8, 1736-1746.

[S5] Xu, L.; Zheng, Q.; Wang, Y.; Jiang, L.; Jiang, J.; Qiu, J. A Pillared Double-Wall Metal-Organic Framework Adsorption Membrane for the Efficient Removal of Iodine from Solution *Sep. Purif. Technol.* **2021**, 274, 118436.

[S6] Zhang, N.; Sun, L.; Xing, Y.; Bai, F. A Double-Walled Thorium-Based Metal−Organic Framework as a Promising Dual-Function Absorbent for Efficiently Capturing Iodine and Dyes *Cryst. Growth Des.* **2019**, 19, 5686-5695.

[S7] Cai, H.; Zeng, G.; You, Z.; Wang, C.; Sun, L.; Bai, F.; Xing, Y. Cu(II) and Zn(II) Frameworks Constructed by Directional Tuning of Diverse Substituted Groups on a Triazine Skeleton and Supermassive Adsorption Behavior for Iodine and Dyes. *Dalton Trans.* **2022**, 51, 5457-5470.

[S8] Zhang, N.; Sun, L.; Bai, F.; Xing, Y. Thorium-Organic Framework Constructed with a Semirigid Triazine Hexacarboxylic Acid Ligand: Unique Structure with Thorium Oxide Wheel Clusters and Iodine Adsorption Behavior. *Inorg. Chem.* 2020, 59, 3964-3973.

[S9] Liu, M.; Cai, H.; Jiang, S.; Xing, Y.; Bai, F. Construction of a Triazine Polycarboxylate Co-MOF with Flexible and Rigid Coordination Arms as well as Excellent Catalytic Reduction and Adsorption Properties. *Dalton Trans.* **2023**, 52, 6773-6781.

[S10] Li, Z.; Yue, Z.; Ju, Y.; Wu, X.; Ren, Y.; Wang, S.; Li, Y.; Zhang, Z.; Guo, X.; Lin, J.
Ultrastable Thorium Metal−Organic Frameworks for Efficient Iodine Adsorption *Inorg. Chem.* **2020**, 59, 4435-4442.

[S11] Huang, J.; Hu, H.; Deng, S.; Cai, S.; Fan, J.; Zhang, W.; Zheng, S. A Ni(II) metal–organic framework with helical channels for the capture of iodine via guest exchange induced amorphization. *New J Chem.* **2022**, 46, 7144-7152.

[S12] Hu, H.; Chen, F.; Zhang, Z.; Liu, D.; Liang, Y.; Chen, Z. Heterometallic Metal-Organic Framework Based on [$Cu_4I_4$] and [$Hf_6O_8$] Clusters for Adsorption of Iodine. *Front. Chem.* **2022**, 10, 864131.

[S13] Falaise, C.; Volkringer, C.; Facqueur, J.; Bousquet, T.; Gasnot, L.; Loiseau, T. Capture of Iodine in Highly Stable Metal – Organic Frameworks: a Systematic Study. *Chem. Commun.* **2013**, 49, 10320-10322.

[S14] Li, Z.; Ju, Y.; Lu, H.; Wu, X.; Yu, X.; Li, Y.; Wu, X.; Zhang, Z.; Lin, J.; Qian, Y. Boosting the Iodine Adsorption and Radioresistance of Th-UiO-66 MOFs via Aromatic Substitution. *Chem. Eur. J.* **2020**, 27, 1286-1291.

[S15] Wang, G.; Huang, J.; Huang, X.; Deng, S.; Zheng, S.; Cai, S.; Fan, J.; Zhang, W. A Hydrolytically Stable Cage-Based Metal–Organic Framework Containing two Types of Building Blocks for the Adsorption of Iodine and Dyes. *Inorg. Chem. Front.* **2021**, 8, 1083-1092.

[S16] Chen, H.; Fan, L.; Zhang, X.; Ma, L. Robust Heterometallic $Tb^{III}/Mn^{II}$–Organic Framework for $CO_2/CH_4$ Separation and $I_2$ Adsorption. *ACS Appl. Nano Mater.* **2020**, 3, 2680-2686.

[S17] Yu, R.; Li, Q.; Zhang, T.; Li, Z.; Xia, L. Zn, O Co-Adsorption Based on MOF-5 for Efficient Capture of Radioactive Iodine. *Process Saf. Environ.* **2023**, 174, 770-777.

[S18] Yang, Y.; Tu, C.; Shi, J.; Zhao, T.; Liu, Z.; Cheng, F.; Luo, F. Highly Stable Cd(II)-MOFs Based on 2,6-Naphthanlenedisulfonate and Bisimidazole Ligands: A New Platform for Selective Detection of $Cu^{2+}$ and Efficient Removal of Iodine. *J. Solid State Chem.* **2021**, 302, 122439.

[S19] Zhang, N.; Xing, Y.; Bai, F. A Uranyl-Organic Framework Featuring Two-Dimensional Graphene-like Layered Topology for Efficient Iodine and Dyes Capture. *Inorg. Chem.* **2019**, 58, 6866-6876.

[S20] Li, M.; Yuan, G.; Zeng, Y.; Yang, Y.; Liao, J.; Yang, J.; Liu, N. Flexible surface-supported MOF membrane via a convenient approach for efficient iodine adsorption. *J. Radioanal. Nucl. Ch.* **2020**, 324, 1167-1177.

[S21] Al Lafi, A. G.; Assfour, B.; Assaad, T. Metal Organic Framework MIL-101(Cr): Spectroscopic Investigations to Reveal Iodine Capture Mechanism. *J. Inorg. Organomet. P.* 2020, 30, 1218-1230.

[S22] Li, L.; Sun, Z.; Yang, A.; Zhang, X.; Zhu, X.; Li, W.; Liu, Y.; Luan, J. A Novel Azo-Linked Polymer Bearing Trifluoromethyl Groups for $I_2$ Capture. *Macromol. Rapid Comm.* **2023**, 44, 2200982.

[S23] Thurakkal, L.; Cheekatla, S.; Porel, M. Superfast Capture of Iodine from Air, Water, and Organic Solvent by Potential Dithiocarbamate-Based Organic Polymer. *Int. J. Mol. Sci.* **2023**, 24, 1466.

[S24] Du, W.; Qin, Y.; Ni, C.; Dai, W.; Zou, J. Efficient Capture of Volatile Iodine by Thiophene-Containing Porous Organic Polymers. *ACS Appl. Polym. Mater.* **2020**, 2, 5121-5128.

[S25] Pan, X.; Ding, C.; Zhang, Z.; Ke, H.; Cheng, G. Functional Porous Organic Polymer with High S and N for Reversible Iodine Capture. *Micropor. Mesopor. Mat.* **2020**, 300, 110161.

[S26] Yang, X.; Duan, L.; Ran, X.; Ran, B.; Yi, S. Two Nitrogen‐Rich Conjugated Microporous Polymers for Efficient Iodine Sequestration and Removal. *J. Polym. Res.* **2022**, 29, 499.

[S27] Sigen, A.; Zhang, Y.; Li, Z.; Xia, H.; Xue, M.; Liu, X.; Mu, Y. Highly Efficient and Reversible Iodine Capture Using a Metalloporphyrin-Based Conjugated Microporous Polymer. *Chem. Commun.* **2014**, 50, 8495-8498.

[S28] Liu, L.; Song, C.; Kong, A. Nitrogen and Sulfur-Enriched Porous Bithiophene-Melamine Covalent Organic Polymers for Effective Capture of $CO_2$ and Iodine. *Mater. Lett.* **2020**, 277, 128291.

[S29] Mohan, A.; Al-Sayah, M. H.; Ahmed, A.; El-Kadri, O. M. Triazine‐Based Porous Organic Polymers for Reversible Capture of Iodine and Utilization in Antibacterial Application. *Sci. Rep.* **2022**, 12, 2638.

[S30] Yang, M.; Shi, W.; Liu, S.; Xu, K. Multifunctional Diphenyl Ether-Based, Cross-Linked Polyisocyanide for Efficient Iodine Capture and $NO_2^-/SO_3^{2-}$ Electrochemical Probing. *Colloid. Surface. A* **2022**, 642, 128680.

[S31] Li, Y.; Li, X.; Li, J.; Liu, W.; Cheng, G.; Ke, H. Phosphine-Based Covalent Organic Framework for Highly Efficient Iodine Capture. *Micropor. Mesopor. Mat.* **2021**, 325, 111351.

[S32] Yan, X.; Yang, Y.; Li, G.; Zhang, J.; He, Y.; Wang, R.; Lin, Z.; Cai, Z. Thiophene-Based Covalent Organic Frameworks for Highly Efficient Iodine Capture. *Chinese Chem. Lett.* **2023**, 34, 107201.

[S33] Jing, L.; Cheng, C.; Wang, B.; Wang, S.; Xie, R.; Xia, H.; Wang, D. Controlled Iodine Phase Transfer of Covalent Organic Framework Membranes for Instant but Sustained Disinfection. *Langmuir* **2023**, 39, 597-609.

[S34] Wu, Z.; Wei, W.; Ma, J.; Luo, J.; Zhou, Y.; Zhou, Z.; Liu, S. Adsorption of Iodine on Adamantane-Based Covalent Organic Frameworks. *Chem. Select* **2021**, 6, 10141-10148.

[S35] Liu, X.; Zhang, A.; Ma, R.; Wu, B.; Wen, T.; Ai, Y.; Sun, M.; Jin, J.; Wang, S.; Wang, X. Experimental and Theoretical Insights into Copper Phthalocyanine-Based Covalent Organic Frameworks for Highly Efficient Radioactive Iodine Capture. *Chinese Chem. Lett.* **2022**, 33, 3549-3555.

[36] Mokhtari, N.; Dinari, M. Developing Novel Amine-Linked Covalent Organic Frameworks towards Reversible Iodine Capture. *Sep. Purif. Technol.* **2022**, 301, 121948.

[S37] Song, S.; Shi, Y.; Liu, N.; Liu, F. C=N Linked Covalent Organic Framework for the Efficient Adsorption of Iodine in Vapor and Solution. *RSC Adv.* **2021**, 11, 10512-10523.

[S38] Song, S.; Shi, Y.; Liu, N.; Liu, F. Theoretical Screening and Experimental Synthesis of Ultrahigh-Iodine Capture Covalent Organic Frameworks. *ACS Appl. Mater. Interfaces* **2021**, 13, 10513-10523.
